# Lipid Horizons: Recent Advances and Future Prospects in LBDDS for Oral Administration of Antihypertensive Agents

**DOI:** 10.1155/2024/2430147

**Published:** 2024-02-19

**Authors:** Preeti, Sharda Sambhakar, Rohit Malik, Saurabh Bhatia, Ahmed Al Harrasi, Renu Saharan, Geeta Aggarwal, Suresh Kumar, Renu Sehrawat, Chanchal Rani

**Affiliations:** ^1^Banasthali Vidyapith, Vanasthali Road, Aliyabad 304022, Rajasthan, India; ^2^Gurugram Global College of Pharmacy, Haily Mandi Rd, Farukh Nagar 122506, Haryana, India; ^3^SRM Modinagar College of Pharmacy, SRMIST, Delhi-NCR Campus, Ghaziabad, India; ^4^Natural & Medical Sciences Research Centre, University of Nizwa, Birkat Al Mouz, Nizwa, Oman; ^5^School of Health Sciences, University of Petroleum and Energy Studies, Dehradun, Uttarakhand, India; ^6^Maharishi Markandeshwar Deemed to be University, Mullana, Ambala 133203, Haryana, India; ^7^Bharat Institute of Pharmacy, Pehladpur, Babain, Kurukshetra 136132, Haryana, India; ^8^School of Medical & Allied Sciences, K. R. Mangalam University, Gurugram, Haryana 122103, India

## Abstract

The lipid-based drug delivery system (LBDDS) is a well-established technique that is anticipated to bring about comprehensive transformations in the pharmaceutical field, impacting the management and administration of drugs, as well as treatment and diagnosis. Various LBDDSs verified to be an efficacious mechanism for monitoring hypertension systems are SEDDS (self-nano emulsifying drug delivery), nanoemulsion, microemulsions, vesicular systems (transferosomes and liposomes), and solid lipid nanoparticles. LBDDSs overcome the shortcomings that are associated with antihypertensive agents because around fifty percent of the antihypertensive agents experience a few drawbacks including short half-life because of hepatic first-pass metabolism, poor aqueous solubility, low permeation rate, and undesirable side effects. This review emphasizes antihypertensive agents that were encapsulated into the lipid carrier to improve their poor oral bioavailability. Incorporating cutting-edge technologies such as nanotechnology and targeted drug delivery, LBDDS holds promise in addressing the multifactorial nature of hypertension. By fine-tuning drug release profiles and enhancing drug uptake at specific sites, LBDDS can potentially target renin-angiotensin-aldosterone system components, sympathetic nervous system pathways, and endothelial dysfunction, all of which play crucial roles in hypertension pathophysiology. The future of hypertension management using LBDDS is promising, with ongoing reviews focusing on precision medicine approaches, improved biocompatibility, and reduced toxicity. As we delve deeper into understanding the intricate mechanisms underlying hypertension, LBDDS offers a pathway to develop next-generation antihypertensive therapies that are safer, more effective, and tailored to individual patient needs.

## 1. Introduction

LBDDS formulation approaches have been given alternative delivery systems for challenging drug molecules possessing poor hydrophilicity. In recent advances in design approaches, numerous drug entities have been developed with potential medicinal significance. However, many of these entities are newly discovered or represent novel chemical compounds. They often fall into the category of high molecular weight and belong to the BCS-II (biopharmaceutical classification system) class, characterized by either high membrane permeability or poor water solubility. This presents a challenge for formulative scientists due to significant variations, first-pass metabolism, and poor bioavailability [1]. In some circumstances, elevation in the molecular configuration may eliminate all these difficulties. LBDDSs represent that innovative approach has demonstrated efficacy in overcoming obstacles related to high membrane permeability, poor water solubility, and other formulation challenges in the development of pharmaceuticals. Encapsulating the entities in lipid/oil can lead to improve the solubilization and absorption of poorly aqueous soluble drugs resulting improved bioavailability [2, 3]. LBDDSs are versatile technologies tailored to meet diverse product needs driven by factors such as disease characteristics, the method of administration, cost-effectiveness, product safety, stability, efficacy, and toxicity. These preparations offer a practical approach to create effective solutions for oral, topical, and injectable (parenteral) delivery [4]. Moreover, ongoing research is exploring the potential of incorporating existing therapeutic compounds, especially those with poor bioavailability and undesirable side effects, into these delivery systems. Antihypertensive drugs, known for their challenging bioavailability and side effect profiles, are actively being studied as promising candidates for integration into such advanced delivery systems [5].

Consequently, LBDDS is an increasingly important field of research that will continue to deliver medicines for better management of hypertension and decrease the toxicity of several drugs by changing the disposition process of drug and keep away from sensitive organs [4]. Also, LBDDSs have been taken the steer because of apparent advantages of higher degree of biocompatibility. The particular attribute of LBDDS is then a function of their size, surface area, surface modification, or encapsulation/solubilization capacity [4].

LBDDSs for oral administration of drugs typically involve a formulation comprising a blend of two or more excipients. These excipients commonly include lipids or oils (triglycerides and partial glycerides), surfactants, and cosurfactants. This liquefied blend serves as an effective medium for the delivery of pharmaceutical entities. While the utilization of lipids and oils for drug encapsulation is not a recent trend, it remains a concept of great promise in pharmaceutical research. Although the approach has been well-established, its auspicious nature continues to inspire ongoing exploration and innovation in drug delivery.

Hypertension is the symbol of all cardiovascular disease. It is an established “silent killer.” According to the eight Joint National Committee On Prevention, Detection, Evaluation, Treatment 2013 (JNU 8), hypertension is a the most commonly occurring disease about 2/3rd of individuals under the age of 60 and almost 1/3rd of young adults [6, 7]. Hypertension stands out as the leading contributor to cardiovascular (CV) disorders, serving as a primary cause of illness and eventual mortality. Persistent hypertension can lead to damage in vital organs such as the kidneys, heart, brain, and blood vessels, contributing to conditions like congestive heart failure (CHF), ischemic heart disorders, strokes, kidney failure, and an increased risk of metabolic syndrome [7].

Hypertension is directly related to arterial blood pressure (ABP), and this relationship is proportional to the combined effects of systemic vascular resistance (SVR) and cardiac output. In simpler terms, elevated blood pressure is closely tied to the balance between the resistance in blood vessels throughout the body and the amount of blood pumped by the heart. When the blood pressure is high, it is an indication that the “blood vessels and heart are actually overburden.” Antihypertensive agents primarily work to reduce cardiac output (CO), systemic vascular resistance (SVR), or both. These agents operate through three main mechanisms as follows:

- Autonomic nervous system-the baroreceptor reflex
- Renin-angiotensin-aldosterone system (RAAS)
- Nitrous oxide hormone: some local hormones liberation from vascular cardiac endothelium.

Throughout hypertension, needful medication treatment is explained as either a persistent diastolic blood pressure more than 90 mmHg or a persistent systolic blood pressure more than 140 mmHg. Hypertension results from altered systemic vascular tones of smooth muscles due to various reasons.

### 1.1. Etiology of Hypertension

Etiology of hypertension is widely spread and occurs due to various reasons involving in one or multiple organ systems of the body [8, 9]:

1. Hypertension occurs due to the renal (kidney) disorders, chronic renal disorders, polycystic disorders, renal artery stenosis, renin producing tumors, and so on
2. Endocrine disorders inducing hypertension: adrenocortical hyper function such as congenital adrenal hyperplasia, Cushing syndrome, hypo and hyperthyroidism, and so on
3. Cardiovascular disorders can induce hypertension through various mechanisms, including an increase in intravascular volume and cardiac output, aortic stiffness, and conditions such as polyarteritis nodosa
4. Neurological conditions: increased intracranial pressure, stress, sleep apnea, psychogenic, and so on

### 1.2. Pathophysiology of Hypertension

**Figure 1:**
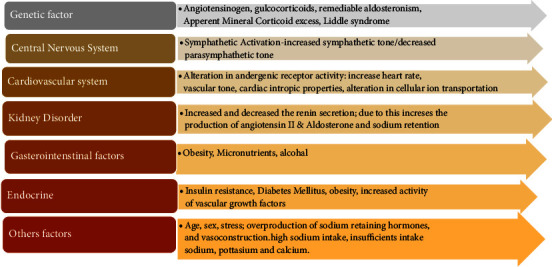
Pathophysiology of hypertension.

Pathophysiology of the hypertension is complex and multifactorial. Kidney plays an important role and we can say that kidney is a target organ of all the hypertension inducing processes. The disease concerns with the several mechanisms of interdependent or independent pathway and interaction of multiple organ systems as shown in Figure 1. Hypertension can be explained by different ways due to their pathogenesis [10]:

1. Hypertension occurs due to vasoconstriction by overactive renin-angiotensin-aldosterone system that retains the sodium and water. Increasing blood volume is also responsible for vasoconstriction inducing hypertension.
2. Atrial natriuretic factors increase secretion promoting the salt excretion when kidney is able to excrete sodium; thus, increased secretion of atrial natriuretic factors increased the total peripheral resistance as of their side effect.

### 1.3. Treatment of Hypertension

**Figure 2:**
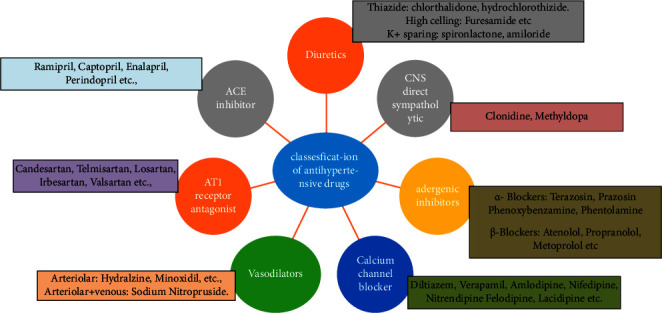
Classification of antihypertensive agents.

Hypertension cannot be eliminated because there is no vaccine developed till now for the prevention of hypertension. Reducing the occurrence of hypertension involves mitigating risk factors associated with its development. These factors include a high dietary intake of sodium and fats, insufficient intake of potassium, obesity, lack of physical activity, and excessive alcohol consumption, among others. In chronic hypertension cases, efforts directed at lifestyle modification can effectively control blood pressure (BP). However, if lifestyle changes are insufficient in achieving adequate BP control, additional interventions may be necessary. Then, antihypertensive agent therapy can be inaugurated with life style modification. Although the availability of more than 75 antihypertensive agents across nine different classes, hypertension in general population is at best insufficient. Treatment of hypertension can be done *via* various groups of antihypertensive drugs as shown in Figure 2.

Antihypertensive agent can be categorized permitting to their mechanism or locations of action. Pharmacological approaches for treating essential hypertension encompass various strategies. The first approach is using diuretic drugs to lower the blood volume, the second approach is antihypertensive agent that acts on the RAAS (renin-angiotensin-aldosterone system), and the third approach is drug reducing the induced cardiac output and SVR or both.

### 1.4. Classification of Antihypertensive Agents

For many of the patients with essential or systemic hypertension, management of hypertension with long-term drug treatment is favorable. There is immense evidence to recommend that antihypertensive agents give protection against the severe complication of the disease. Luckily, a number of hypertensive agents are available to proficient management of hypertension disease. The classification of antihypertensive agents is shown in Figure 2. Choosing the right drug for therapy and closely monitoring its effects provide the best strategy to reduce the mortality, morbidity, and complications associated with hypertension [11].

#### 1.4.1. Diuretics

**Figure 3:**
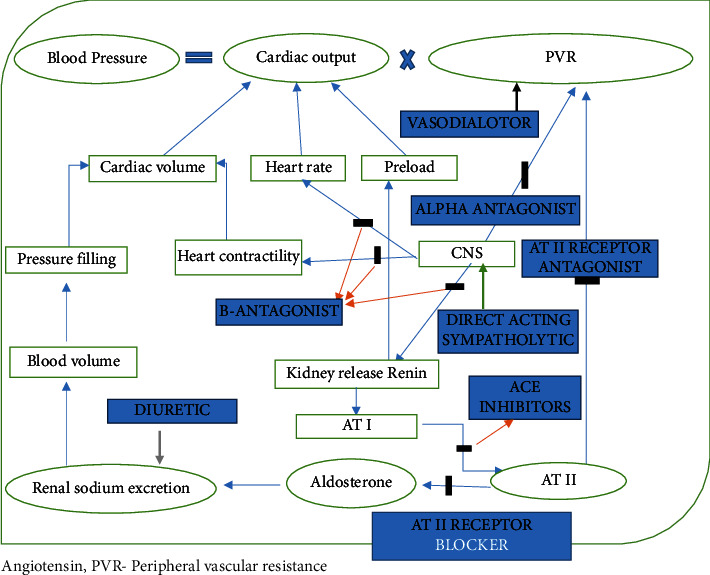
Mechanism of action of antihypertensive drugs.

The first approach for the hypertension management is use of diuretic drugs to reduce the blood volume. Diuretic drugs were introduced nearly five decades ago and used for hypertension treatment; the most commonly prescribed drugs for hypertension are thiazide diuretics. Diuretic drugs effectively reduce the blood pressure and decrease the risk of cardiovascular outcomes due to hypertension. Diuretic drugs were widely used either as a monotherapy or in combination with other classes of antihypertensive agents. According to the current guidelines for adults, like JNC 8 (2014), European Society of Hypertension/European Society of Cardiology (ESH/ESC 2013), National Institute for Health and Care Excellence (NICE 2011), and Canadian (2014), thiazide diuretics are recommended as the first-choice medication for starting antihypertensive therapy to manage hypertension [12]. Thiazide diuretics act on the distal convoluted tubule to obstruct the reabsorption of Na+ and Cl-, thereby reducing extracellular and plasma fluid volume. This reduction leads to a decrease in cardiac output (CO) as shown in Figure 3. Diuretic drugs in combination with other drugs that are acting on the renin-angiotensin-aldosterone drugs such as ACE inhibitors enhance the effectiveness of diuretic drugs by obstructing responsive hyperreninemia. Thiazide drugs may be less effective in individuals with renal insufficiency, in the presence of excessive salt intake, and for those patients concurrently using NSAIDs [11]. Studies prove that long-term treatment *via* thiazide diuretics defends against the osteoporosis condition because of their hypercalcemic effect [13].

#### 1.4.2. Adrenergic Inhibitor Drugs

These drugs are used to control hypertension because inadequate sympathetic activity plays a significant role in the pathogenesis of hypertension. Epinephrine and norepinephrine are sympathetic neurohormones that produce cardiovascular action by triggering the sympathomimetic (*α* and *β*) receptors. So, antagonism of particular receptors by therapeutical assets decreases the PVR, cardiac output, or both.

*β*1 adrenergic inhibitor drugs (atenolol, metoprolol, and so on) reduce the heart rate and contractility; O_2_ demands ultimate cardiac output as shown in Figure 3, and these drugs are used either as monotherapy or in combination with other drugs, most commonly with diuretics or vasodilators for the best management of hypertension or other cardiac condition.

Equally, *α*1 antagonist drugs (prazosin, terazosin, and so on) also have antihypertensive efficacy similar to diuretics, *β*1 inhibitors, ACE inhibitors, and calcium channel blockers (CCBs). *α*1 blocker drugs have reduced preload, afterload PVR, and also show the vasodilation action. *α*1 antagonist drugs can be used as a monotherapy or in combination with CCB, *β*1 blockers, or diuretics for hypertension management [12].

#### 1.4.3. Direct Acting Sympatholytic Drugs

Direct acting CNS sympatholytic drugs (*α*2-selective adrenoceptor agonist) are the oldest class of antihypertensive drugs (methyldopa and clonidine). Activation of *α*2-adrenergic receptors in the vasomotor center leads to decreased sympathetic nerve activity, resulting in a reduction in peripheral vascular resistance (PVR). These drugs show the best result when used in combination with diuretic drug or sodium intake restriction for hypertension management [11].

#### 1.4.4. Angiotensin Converting Enzyme (ACE) Inhibitors

ACE inhibitor drugs (ramipril, captopril, lisinopril, and so on) as their name implies prevent the formation of angiotensin II (AT II) by inhibiting the enzyme activity as their mechanism shown in Figure 3 and relive in vasoconstriction. ACE inhibitor drugs also interfere by blocking the tissue renin-angiotensin mechanism [14]. Angiotensin inhibition reduces the production of aldosterone, and long-term therapy with ACE inhibitor drugs lowers the plasma aldosterone level to the normal baseline values. These drugs may also suppress the sympathetic nervous system activity and reduces the secretion of endothelin. These drugs directly or indirectly improve vascular distension and augment endothelial function. ACE inhibitor drugs commonly accepted in the management of hypertension and CHF also these drug gives renal protection because of favorable expansion of the afferent capillaries which decreases the intra glomerular pressure. ACE inhibitor drug is also a drug of choice for patients with diabetic's mellitus because these drugs improve to be insulin sensitive. These drugs prove beneficial in patients with renal dysfunction and in those with heart failure ACE inhibitor drugs which are choice of drugs for these candidates [12].

#### 1.4.5. Angiotensin II Receptor Antagonists

At present time, for the treatment of hypertension, there are a number of active angiotensin II (AT II) receptor antagonists (candesartan, losartan, and so on) which are available representing therapeutic effect in the interference of the renin-angiotensin-aldosterone system to lower the BP [15, 16]. These drugs block the action of AT II on the heart, blood vessels, and adrenal cortex. Similarly, drugs like ACE inhibitors provide relief by counteracting vasoconstriction, vascular and myocardial hypertrophy, and inhibiting the secretion of aldosterone. Angiotensin antagonist's drugs are effective against the hypertension as monotherapy or in combination with diuretics drugs, and these are available in fixed dose combination with thiazide diuretics.

#### 1.4.6. Vasodilators

Vasodilator drugs (hydralazine and minoxidil) directly decrease the PVR, i.e., desirable in the management of hypertension. The mechanism of action for vasodilators drugs is to encourage mitigation of the cardiac smooth muscles in the restricted veins and artery. These drugs are mostly used in combination with adrenergic receptor blockers and diuretic drugs [11]. Vasodilator drugs are effectively used for the management of resistant hypertension with long-term therapy [17].

#### 1.4.7. Calcium Channel Blocker

Calcium channel blockers (CCBs) were developed to diagnose arrhythmia or angina pectoris and now are popular as treatment of hypertension. From a pharmacological aspect, CCBs are classified into dihydropyridines such as isradipine and amlodipine and benzodiazepine such as diltiazem, and phenylalkylamines such as verapamil [18]. Instead, CCBs are also categorized into rate liming agents/drugs (verapamil and diltiazem) and rate accelerating agents/drugs (isradipine and so on) [19]. Dihydropyridine CCBs are strong vasodilators although diltiazem and verapamil have moderate vasodilation action. Dihydropyridine drugs encourage AV conduction and heart contractility whereas verapamil and diltiazem suppress them. The effects of calcium channel blockers (CCBs) are primarily mediated by the blockade of L-type Ca2+ channels, influencing tissue events and lifespan accordingly. CCBs as an antihypertensive drug effective as single drug therapy or in combination using other categories of antihypertensive agent such as *β*-blockers drugs are effectively be used with dihydropyridine drugs.

## 2. Shortcomings of Antihypertensive Agents

Major challenge that is associated with the antihypertensive agents administered by oral route is their poor bioavailability. Poor bioavailability of drugs has a consequence because dose of such drugs recommended is high because only a small portion of the drug spreads into the systemic circulation and is accountable for inducing adverse effect. Many of these antihypertensive agents exhibit poor bioavailability, often less than 50%, as indicated in Table 1. This is attributed to various reasons.

### 2.1. Poorly Aqueous Solubility

These factors significantly impact the absorption and bioavailability of drugs, particularly BCS-II and IV class drugs, which encounter this issue. In recent review studies, it was claimed that almost 40% of the newly discovered drugs and chemical molecule entities suffer from the poor aqueous solubility [56]. This review comprises numerous reports on poor availability of drugs due to poor aqueous solubility. It has been evaluated that if the dissolution proportion of drugs is adequately slower than the absorption proportion, then drug dissolution is considered as rate determining step (RDS) for the absorption process. Therefore, it is relevant that drugs with low intrinsic solubility and slow dissolution rates exhibit poor systemic availability [57].

### 2.2. Inappropriate Lipophilicity (Partition Coefficient)

Partition coefficient is a major contributing factor for prediction of membrane permeability, as it influences the penetration of drug across the biomembrane. Significant lipophilicity is requisite to ease the partitioning of a drug molecule into lipoidal biomembrane so that it can penetrate and easily be available in the systemic circulation. Drugs with partition coefficient values (log P) in the range of 1–3 exhibit significantly better passive absorption through membranes, while others drugs whose values outside the given range are greater than or less than have poor transportation characteristics [58].

### 2.3. High Molecular Weight of Drug

Most orally administered drugs are absorbed *via* passive diffusion to attain high drug concentration in plasma in order to produce supreme therapeutic effect. Molecular weight and size of the drug are the most important factors on directing their permeation capability across biomembrane, and hence high molecular weight drugs are not capable to cross such biological barrier passively. A *Rule of Five* discussed the influence of main three parameters on the permeation of a drug molecule including surface polarity, lipophilicity, and molecular weight and size [59].

### 2.4. First-Pass Metabolism

This occurs when drugs undergo extensive metabolism before reaching sufficient plasma concentrations, resulting in low bioavailability. In the case of antihypertensive agents with poor bioavailability (<50%), the primary contributing factor is hepatic first-pass metabolism, as illustrated in Table 1 for oral administration. Orally administered drugs are absorbed from the GIT and transported to the liver and kidney, where it gets metabolized. As an outcome, the availability of drug in systemic circulation is greatly reduced [60], thus affecting drug concentration reaching its considered target site. Due to inadequate plasma concentrations, the overall availability of the drug is significantly reduced. The primary site that is responsible for the first-pass metabolism is hepatic enzyme, GIT lumen enzyme, gut wall, and bacterial enzyme [61].

### 2.5. Drug Degradation in GIT Lumen

Drug substances used as medicines have diverse molecular structures and are consequently prone to many and variable degradation pathways because of gastric acidic pH, chemical interaction happened in GIT, enzymatic mortification in the GIT of drug as GIT is the pivot of enzymes, and degradative action of these enzymes leading deterioration of chemical structure of the drugs and reducing their absorption eventually fallen in systemic availability [62].

### 2.6. Food Interaction

Food also influences the drug bioavailability. Intake of food with drugs exerts numerous changes, such as increased residence time, increased gastric motility, gastric pH changes, and also increased perfusion rate to GI mucosa and liver, so these changes can influence the absorption and drug pharmacokinetic to a larger extent [63]. Orally administered drugs may be affected in several ways by food [64]. For, e.g., several poorly water-soluble drugs, including griseofulvin and certain antihypertensive agents, demonstrate increased bioavailability when taken with food. Conversely, other drugs like isoniazid and rifampicin, used in the treatment of tuberculosis and leprosy, exhibit poor systemic availability when orally administered with food.

### 2.7. Drug-Efflux Pump

Factors such as P-glycoprotein (P-gp) play a significant role in modifying the pharmacokinetics of various drugs. The presence of P-gp in the liver, intestine, and kidney contributes to a reduction in drug absorption in the gastrointestinal tract (GIT) and an increase in drug elimination. The combined action of P-gp and CYP3A4 in the gut wall works synergistically to regulate the absorption of substrates, providing CYP3A4 with multiple opportunities to metabolize compounds in the gut.

### 2.8. High Gastric Emptying Rate

Inadequate time in the gastric for absorption is a common cause as it is a prominent absorption site for various drugs. Hence, short gastric transit time results in low bioavailability of these drugs [64, 65]. It is broadly accredited that the gastric emptying transit time of a dosage is an accountable factor for the drug absorption variation amongst individuals. The factors that affect the gastric emptying include gender, age, fed or fasted state, nature, volume and composition of meal, viscosity, temperature or caloric content of the meal, stress, disease state, and several drugs affecting the emptying rate [66–68].

## 3. Oral Bioavailability Enhancement Approaches

**Figure 4:**
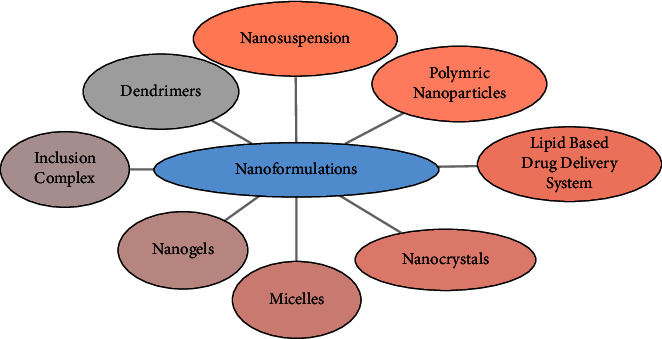
Classification of nanoformulation.

Various approaches have been applied to enhance the bioavailability of aqueous-insoluble or hydrophobic drugs, including molecular optimization, micronized formulation, and novel drug delivery systems such as microemulsion and microsponge; cosolvency; complexation; nanoformulations such as polymeric nanoparticles, micelles, dendrimers, and nanogels; and lipid-based formulation such as self-nanoemulsifying drug delivery system (SNEDDS). Nanodelivery systems or nanoformulations have emerged as a means to enhance efficacy, reduce systemic side effects, and improve patient compliance. Examples include nanosuspensions and nanoemulsions and lipid-based formulation (solid self-nanoemulsifying drug delivery system, liposomes, and so on). Nanoformulation has presented the beneficial evident of enhanced absorption of drugs, prominent to enhance the bioavailability. Nanoformulations for the oral delivery system have gained remarkable drive in recent few years. These formulations transport the active pharmaceutical ingredients (APIs) in a reduced nanosize, leading to an increase in the effective surface area. This enhancement in surface area results in improved dissolution rates of the active substances, thereby significantly increasing the bioavailability of poorly water-soluble drugs. As an innovation, pH reactive constituents offer a substitute release mechanism. Commercially, nanosystems are available as tablets, capsules emulsions, and suspension. These formulations were commercialized in the world all over and mostly manufactured in different manufacturing unit of the USA. These formulations are available for the lipid regulation, kidney disease, diabetes treatment, and so on. Beside from the accepted nanoformulation that is available in the market, there are several go through preapproval or clinical trials and they are all set to arrive into the market globally [62–74]. Nanoformulations are reported to enhance cellular acceptance, elevate biological activity, and increase in vivo bioavailability by modulating physicochemical properties that facilitate drug release and its biological behavior. In addition, they provide protection to encapsulated drug substances, improving stability in the gastrointestinal tract, and offer the advantage of dose reduction. If we do comparison amongst bioavailability enhancement technique, nanoformulation is more efficient and simply taken up by the tissues of the lymphatic system to bring the drugs straight into the targeted site of action [75, 76]. The classification of nanoformulation is shown in Figure 4 and their detailed description is given in Table 2.

### 3.1. Lipid-Based Drug Delivery System (LBDDS)

**Figure 5:**
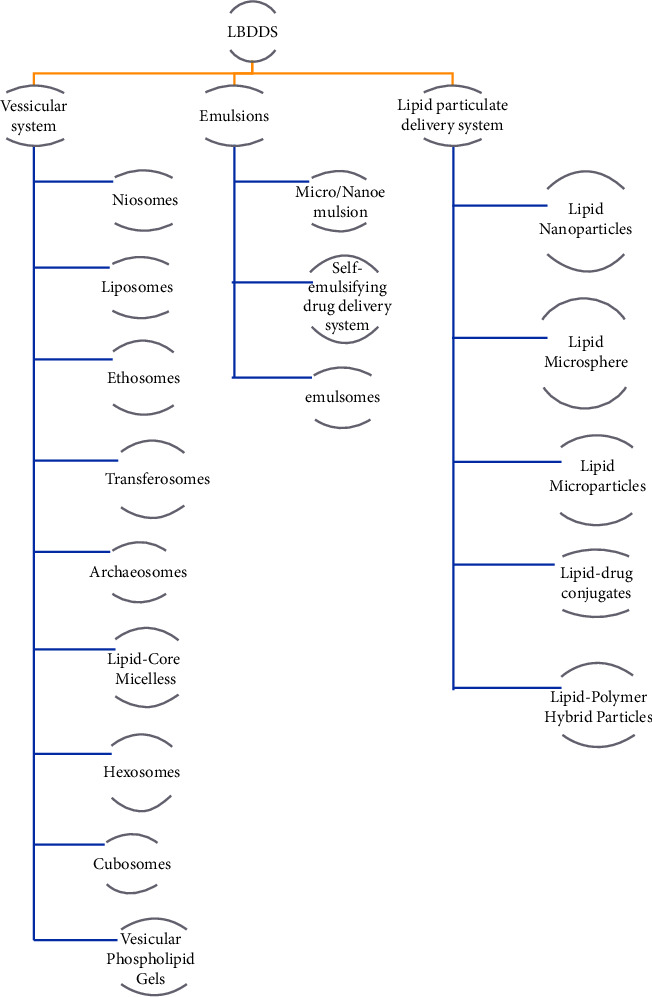
Classification of the lipid-based drug delivery system.

The bioavailability of many drugs, especially those with poor water solubility, can be significantly improved through lipid-based drug delivery systems. These systems utilize lipid formulations to enhance drug solubility, stability, and absorption, thus increasing therapeutic efficacy. This approach is particularly useful for delivering both hydrophobic and hydrophilic entities efficiently. A thoughtful selection of lipid/oil vehicles, formulation development strategies, and rationale are pivotal in guiding the success of LBDDS design. By utilizing various lipid excipients, surfactants, and techniques like nanoemulsions, SLNs, NLCs, liposomes, and micelles, researchers and pharmaceutical companies can develop efficient and targeted drug delivery systems that address the challenges associated with poorly water-soluble drugs as shown in Figure 5 [4, 116].

#### 3.1.1. Classification of Lipid-Based Drug Delivery System

*(1) Vesicular System*. Lipid-based drug delivery systems (LBDDSs) encompass various vesicular systems that utilize lipids as carriers to enhance drug solubility, stability, and bioavailability. These vesicular systems include the following [5, 116]:

 
*Niosomes.* Niosomes are nonionic surfactant vesicles that resemble liposomes but are composed of nonionic surfactants instead of phospholipids. They have a similar structure to liposomes and can encapsulate hydrophilic and hydrophobic drugs. Niosomes offer improved drug stability and prolonged drug release.
 
*Liposomes*. Liposomes are spherical vesicles composed of lipid bilayers, closely resembling cell membranes. They can encapsulate both hydrophilic and hydrophobic drugs due to their aqueous core and lipid membrane, respectively. Liposomes offer controlled drug release, protection of labile drugs, and targeted delivery through surface modifications.
 
*Ethosomes*. Ethosomes are similar to liposomes but contain a higher concentration of ethanol as an enhancer to improve skin permeation. Ethosomes are particularly useful for enhancing transdermal delivery of hydrophobic drugs.
 
*Transfersomes*. Transfersomes are ultradeformable vesicles designed to enhance drug penetration through the skin. They contain edge activators that increase vesicle flexibility, allowing them to squeeze through narrow pores in the stratum corneum for transdermal drug delivery.
 
*Archaeosomes*. Archaeosomes are vesicles composed of archaeal lipids, which are unique in structure and composition compared to traditional phospholipids. Archaeosomes can encapsulate a wide range of drugs and have been explored for their potential in vaccine delivery.
 
*Lipid-Core Micelles*. Lipid-core micelles are formed by amphiphilic molecules, with a lipid core surrounded by hydrophilic outer layers. These micelles can solubilize hydrophobic drugs in their core and offer improved drug stability and bioavailability.
 
*Cubosomes and Hexosomes*. Cubosomes and hexosomes are specialized vesicular systems with cubic and hexagonal liquid-crystalline structures, respectively. They offer unique advantages in encapsulating and delivering both hydrophobic and hydrophilic drugs.
 
*Vesicular Phospholipid Gels*. Vesicular phospholipid gels are semisolid formulations that combine lipids and gelling agents. They can enhance drug penetration through the skin and provide controlled release.

These vesicular systems leverage the properties of lipids to create versatile drug delivery platforms that cater to different drug types, administration routes, and therapeutic goals. They are particularly effective for overcoming challenges associated with poor drug solubility, rapid degradation, and inefficient delivery to target sites.

*(2) Emulsion System*. Lipid-based drug delivery systems (LBDDSs) can also include emulsion systems, which utilize lipid components to create stable emulsions for improved drug delivery. Here are some classes of emulsion systems within LBDDS [117, 118]:

 
*Micro/nanoemulsions*. Micro/nanoemulsions are transparent, thermodynamically stable systems formed from oil, water, and surfactants. Their small droplet size allows for spontaneous drug dissolution and enhanced bioavailability. Microemulsions can be used for both hydrophobic and hydrophilic drugs.
 
*Self-Emulsifying Drug Delivery Systems* (SEDDSs): SEDDSs are emulsion-based systems that spontaneously form fine oil-in-water emulsions or microemulsions when introduced to aqueous media, such as the gastrointestinal tract. SEDDSs enhance oral absorption of poor water-soluble drugs.
 
*Emulsomes.* Emulsomes are a hybrid of liposomes and emulsions. They consist of lipid bilayers surrounding an oil core, providing the benefits of both systems. Emulsomes can encapsulate hydrophilic and lipophilic drugs simultaneously.

Each class of the emulsion system offers distinct advantages for delivering hydrophobic and hydrophilic drugs. Emulsions provide a versatile platform for encapsulating drugs with different physicochemical properties, and their properties can be tailored through careful selection of lipid excipients, surfactants, and formulation parameters.

*(3) Lipid Particulate Delivery Systems*. These systems used lipid-based particles to enhance drug delivery. Here are some classes of lipid particulate delivery systems within LBDDS [5, 116–119]:

 
*Lipid Nanoparticles (LNPs)*. Lipid nanoparticles are colloidal carriers with sizes typically in the nanometer range. This class includes solid lipid nanoparticles (SLNs) and nanostructured lipid carriers (NLCs). SLNs consist of a solid lipid core, while NLCs incorporate a mixture of solid and liquid lipids. LNPs can encapsulate hydrophobic and hydrophilic drugs, improving solubility and bioavailability.
 
*Lipid Microspheres*. Lipid microspheres are larger particles than nanoparticles, often ranging from 1 to 100 micrometers. They offer controlled drug release and can be designed to float in the stomach, providing prolonged gastric retention for drug absorption.
 
*Lipid Microparticles*. Lipid microparticles are solid lipid particles that can encapsulate hydrophobic drugs. They can provide sustained release and protect drugs from degradation.
 
*Lipid-Drug Conjugates.*Lipid-drug conjugates involve attaching drug molecules to lipid moieties. These conjugates can self-assemble into nanoparticles, improving drug solubility and delivery.
 
*Lipid-Polymer Hybrid Particles*. Lipid-polymer hybrid particles combine the advantages of lipids and polymers. These particles can encapsulate drugs within a lipid core and use a polymer shell for stability and controlled release.

These lipid particulate delivery systems offer various advantages in terms of drug solubilization, controlled release, and enhanced bioavailability. The choice of system depends on the drug's physicochemical properties, intended administration route, and therapeutic goals.

#### 3.1.2. Mechanism of Action of LBDDS

**Figure 6:**
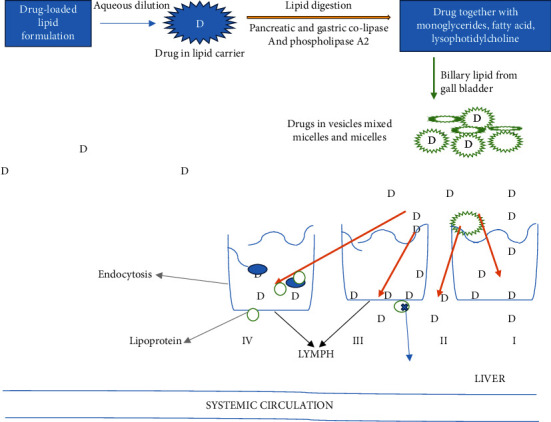
Mechanism of drug transportation *via* the intestine from LBDDS.

*(1) Drugs Absorption via Lymphatic System*. LBDDS increases the absorption through the GIT via accelerating dissolution process and expedite the development of solubilize phases via deduction of particle size up to the microscopic level [118, 119]. From the lipid/oil carrier, the transport of drugs into systemic circulation via the lymphatic system shows a significant part. The main adventurousness of LBDDS drug transportation is apprehension of first-pass drug metabolism and targeting toward specified condition or disorder. Promising mechanisms of LBDDS that influence any entities/drug fascination and disposition as well as bioavailability afterward orally administered is summarized in Figure 6 [120–122].

The adequate mechanism includes the following:

- Improved membrane fluidity *via* facilitating transcellular transportation
- Opening the constricted junction following paracellular transport
- Surfactant used in LBDDS increased the intracellular absorption and duration because of retardation of CYP450 and P-glycoprotein
- Increased lipoprotein and chylomicron production due to stimulation of fatty acid

**Figure 7:**
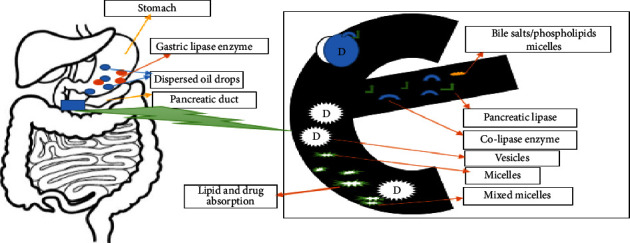
Drug solubilization and lipid digestion in the small intestine.

*(2) Drug Solubilization and Lipid Digestion in Small Intestine (SI)*. When LBDDS are administered orally, digestive lipase, i.e., secreted by the gastric chief cell, commences the digestion of exogenic dietary triglycerides (TGs). In SI, TGs are fragmented into fatty acid (FA), mono/diglyceride, and via lipase, i.e., present in pancreatic juice acted jointly using cofactor lipase 203 [122]. Pancreatic phospholipase breaks down biliary-derived phospholipids and those derived from the formulation through hydrolysis, resulting in the production of fatty acids (FAs) and lysophosphatidylcholine. The incidence of extracellular lipid in the SI promotes secretion of endogenic biliary lipid from cholecyst, along with phospholipids, bile salts (BS), and saturated fatty acid. Previously formed lysophospholipids, monoglycerides, and fatty acids (products of lipid digestion) are subsequently incorporated into colloidal structures, including micelles and uni- and multi-lamellar vesicles, in the presence of bile salts (BS). The absorptive and solubilization capability of SI for lipid digested products and drug/entities increased considerably as these made lipid metabolite afterward hydrolysis.  Figure 7 illustrates the drug solubilization and lipid ingestion method in SI [123].

#### 3.1.3. Lipid-Based Formulation (LBFs) Classification System

This classification was first introduced by Pouton in 2000 and adjusted to encompassed type IV classification in 2006. LBFs are categorized into four types as displayed in Table 3.

#### 3.1.4. Formulation Components

**Figure 8:**
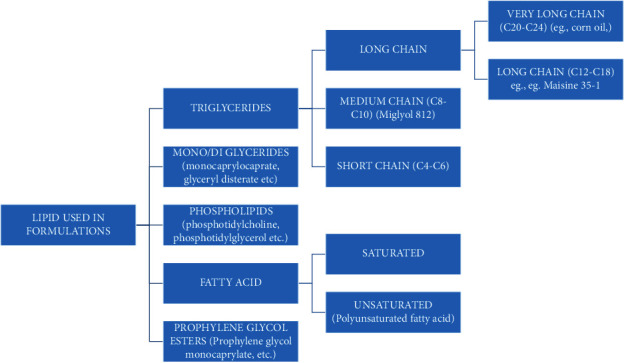
Lipid classification used in formulation development.

*(1) Lipid/Oil*. Lipid or oil is one of the most important components of LBDDS. For antihypertensive drugs, choice of oil/lipid is an important consideration [124, 125]. Lipids or oils classification is based on their physical assets including strength of saturation: saturated and mono/polyunsaturated as illustrated in Figure 8. This classification is essentially valuable for predicting how the digestible fatty acids, which influence the serum lipid-elevating effect of fat, can be attributed to its saturated fatty acid content. The FA has been classified on its saturation level as very long chain (20 : 0–24 : 0), long chain (12 : 0–18 : 0), medium chain (8 : 0–10 : 0), and short chain (4 : 0–6 : 0). In the design and development of LBDDS, medium- and long-chain triglycerides lipids/oils with variable degree of saturation or hydrolysis have been widely used [126–130]. Epidemiological studies of saturated FA content have manifested a low occurrence of coronary heart diseases in the population even though they ingest a diet that is high in. Increased dietary consumption of the polyunsaturated fatty acid (PUFA) is associated with decrease the risk of cardiovascular damage and atherosclerosis. Long-chain PUFA acts as antihypertensive agent and decreases the possibilities for unfavorable cardiovascular disease by increasing the production of vasodilator prostaglandins. Long chain PUFA also shows their activity as inhibitor of angiotensin converting (AC) enzyme [129, 130].

*(2) Classification of Lipid on the Basis of Their Sources*.

 
*Natural Product Oils*. There are several natural product oils that were derived from plant sources and further treated to eradicate filths or to isolate the numerous fractions of the originals derived sources and are appropriate for use in encapsulation of oral formulation products. Naturally, oils and fats are encompassed mixture of triglycerides which contain fatty acid of various degrees of unsaturation and chain length. The melting points of oils increase with the chain length of fatty acid and decrease with the degree of unsaturation, which also increases susceptibility to oxidation. Isolation of natural oils into glycerides fraction is further used to prepare excipients that maximize desired physical and drug fascination promising properties which reduces susceptibility to oxidation [130, 131].
 
*Semisynthetic Lipids*. Various semisynthetic lipid liquids and thermal softening (melt at 26–700°C and in room temperature existed as a waxy and semisolid form) solid excipients were synthesized by chemically combining glycerides derived from plants oils or medium chain saturated fatty acid with polar chemical molecules commonly used as a pharmaceutical component for the oral formulation development system. These lipids were finding their application as a vehicle for drug solubilization, wetting agents, surfactant, and cosurfactant in various SEDDS or SMEDDS formulation. These lipids were well-suited for both the hard and soft HPMC capsules [130].
 
*Synthetic Lipid*. There are several fully synthetic polymeric and monomeric semisolid and liquid components. Many out of them are nontoxic and glycolic in nature and they are used as a vehicle for poorly aqueous soluble drugs. They can be used alone or in combination with others lipids to enhance the solubilization of formulation such as polyethylene glycol, propylene glycols, and poloxamers [130].

*(3) Emulsifiers*. After the lipid, another vital excipient for LBDDS is surfactant and is responsible for the necessary emulsifying properties attribution. Surfactants being amphipathic can easily solubilize maximum amount of water insoluble drug compounds. Surfactant obtained from the natural source is found as much nontoxic or safe than the synthetic one. In formation development of LBDDS, hydrophilic-lipophilic balance (HLB) value of the emulsifier as shown in Table 4 provides main information on its potential utility [130–132]. The generally used emulsifiers include the nonionic properties with high HLB values like polysorbate ethoxylated polyglycolized glycerides 80 (i.e., Tween 80). Nonionic emulsifiers are well-advised as they are not harmful or nontoxic than the ionic ones. In LBDDS, the proportion of emulsifiers usually should be ranged in 30–60% w/w because higher concentration of emulsifier or surfactants may be causing the irritation to the GIT mucous [130].

*(4) Coemulsifiers/Cosolvents*. When developing optimized self-emulsifying LBDDS formulations, a moderately high concentration (<20%) of surfactant is required. Additionally, a coemulsifier is added to enhance self-emulsification. The presence of cosurfactants helps reduce interfacial stress. Other organic solvents including propylene glycol, polyethylene glycol, and ethanol are appropriate for delivery by oral route and they allow the dissolution of large quantity of either the water-soluble surfactant or the drug in the lipid/oil carrier. Second, alcohol and other volatile nature cosolvent have the drawback of evaporating into the shell of the hard and soft gelatin. However, mostly the alcohol-free formulations have been designed, but their water insoluble drug dissolution capacity may be limited [130–134].

*(5) Emulsifier: Coemulsifier Ratio*. The emulsifier: coemulsifier ratio has been establishing an important aspect for improving the phase behaviors, i.e., size and emulsification position part. The typical orientation is to elect formulation component with the low emulsifier's concentration for oral route of administration [130, 131].

#### 3.1.5. Formulation Approaches for LBDDS

For the preparation of LBDDS, there are several techniques used as discussed in the following.

*(1) Spray Drying*. This technique is also similar to the spray congealing but differs in air temperature inside the atomizing compartment. In this method, drug in organic solvent/water (drug solution) is sprayed into a hot air chamber, wherein the solvent or water evaporates giving rise to drug micro/nanoparticles. Through this progression, the lipid excipients can also be used. Lipid excipients (monoglycerides, triglycerides, and polyunsaturated fatty acid) improve the drug release of drug substances [4, 135, 136].

*(2) Spray Congealing*. This technique is also known as spray cooling. In this technique, liquefied lipid or oil is drenched into conserving cavity, in contact with cool air, solidifying into sphere-shaped particles. These particles were collected from the bottom of the chamber and further which can be compressed into a tablet or filled into hard gelatin capsules. For the spray cooling process, ultrasonic atomizers are most commonly used to produce solid particles. The factor affecting the solidification of the particles is melting point of the excipient, and freezing air temperature in chamber allows the solidification of droplets and viscosity of the formulation [4].

*(3) Absorption of Solid Carrier*. This technique is one of the simplest methods in this liquid lipid preparation such as nanoemulsion, nanoparticles, and self-nanoemulsifying liquid and is fascinated onto solid carrier like Aerosil 200, cetyl palmitate, and silicon dioxide. During the process, lipid liquid preparation is added into a carrier by mixing using suitable methods such as ultrasonication and homogenization. These solid carriers must be chosen such that it had a higher capability to adsorb the lipid liquid formulation and after absorption have a good flowing property. There are several antihypertensive drugs such as nitrendipine and valsartan which were efficaciously incorporated into solid carrier to improve the bioavailability and avoid the first-pass metabolism of drug. This method has several advantages including content homogeneity and extraordinary lipid coverage [137–139].

*(4) Supercritical Fluid-Based Method*. This technique uses lipid material for coating the drug powder to produce dispersion (solid). In this technique procedure, the drug and lipid excipients are dissolved into in solvent and SCF (supercritical fluid) such as carbon dioxide, by uplifting the pressure and temperature. This coating procedure is expedited by a steady decrease in temperature and pressure in order to decline the solubility of the coating materials in the fluid and later precipitate the drug particles to form the coat [139–142].

*(5) Palletization*. This technique is also known as melt granulation which is basically based on the transformation of the powder drug mixture into pellets or granules [143–145]. In the process, a melted binder is drenched into the drug powder mixture in presence of high constraint mixing, and alternatively the meltable binders is mixed with drug powder and due the abrasion between the particles during high constraint forces the binder melts. The liquefied binder forms liquid bridges between the particles and formulate small particles which is further converted into small pellets under precise circumstance. Depending on the powder refinement, liquid binder can be used about 15%–25%. These factors considered throughout the processing are particle size of binding agent, intercourse timing, impeller rotating speed (rpm), and viscosity of melted binder [146]. This technique is used mostly to form self-micro/nanoemulsifying drug delivery system.

#### 3.1.6. Characterization of LBDDS

*(1) Appearances*. This can be evaluated in transparent glass container or graduated cylinder for its uniformity and color appearance [4, 147].

*(2) Color, Odor, and Taste*. These are important characteristics for the orally administered drug delivery systems. Active substances taste variation is occurring due to changes in crystals habit, particle size, and so on. Changes in these characteristics indicate the chemical instability [5, 148, 149].

*(3) Density*. Density and specific gravity of the LBDDS are essential parameters. A reduction in density often specifies the entrapment within the construction of the preparation. Measurement of density at a specified temperature can be achieved by hydrometers [148].

*(4) pH Value*. pH value of lipid liquid formulation should be engaged at a specified temperature condition *via* pH meter and later settle down evenness has been obtained, to minimize “pH drift” and coating with suspended particles on electrode surface. To stabilize the pH of the formulation, electrolyte should not be put into the external phase of the lipid formulation because these electrolytes will interrupt the physical stability of the formulation [148].

*(5) Self-Dispersion and Sizing of Dispersion*. Lipid-based formulation having evaluation of dispersion rate and subsequent particle size is desirable and consequently consideration has been specified to determine the dispersion rate. For the particle size measurement, the optical compound microscope can be used for the particles within micron. Particle size can be analyzed by the particle size analyzer [5, 150].

*(6) Globules Size*. It is the globules size distribution for micro/nanoemulsion. Other lipid-based formulation can be determined by the either light scattering (dynamic light scattering) or electron microscopy technique (scanning electron microscopy or transmission electron microscopy) [151].

- *Dynamic Light Scattering (DLS)*. DLS is used for the physicochemical characterization of nanoformulations such as size, structure, and shape, and aggregative state conformation on molecular level can be evaluated by using DLS. DLS is working on the principle of Rayleigh scattering, induced from the Brownian movement of particles of a dimension much lesser than the incident light wavelength at a static angle of scattering. DLS is the ability to measure diluted specimen samples.
- S*canning Electron Microscopy (SEM)*. SEM uses light source and glass lenses irradiate the sample to produce enlarged images. Electron microscope usages augmented electron beam and electromagnetic and electrostatic lenses to produce imaginings of considerably advanced resolution that basically depends on the much shorter wavelength of electron beam than visible light photon. In SEM, electron beam incident on the sample surface and produced signals reflect the atomic composition and morphological information (size distribution, shape, and particle size) of the specimen surface.
- *Transmission Electron Microscopy (TEM)*. This is one of the most frequently used technique for the characterization of lipid-liquid formulations. TEM produces chemical information and direct image or nanoformulation at a 3D resolution down to the level of atomic dimension. In high spatial resolution of the TEM, the structural and morphological analysis of the lipid formulation is improved.

Both the techniques SEM and TEM are used to expose the size and shape diversification of formulation and also the amount of dispersion and aggregation. TEM is most commonly used as having an advantage over SEM because it provides a better longitudinal resolution and ability for supplementary analytical measurement [149–151].

*(7) Zeta Potential (Surface Charge)*. It is determined by the using analyzer of zeta potential of the sample preparation. It gives information regarding the repulsive forces between the globules and particles. To obtain a stable lipid liquid nanoformulation by preventing coalescence and flocculation of the nanoglobules, zeta potential should typically reach values above 30 mV [151].

*(8) Viscosity Measurement*. Viscosity of different lipid liquid formulation can be measured by using Brookfield type rotary viscometer at different temperatures. The samples are to be immersed before measurement, and the sample temperature should be maintained at 370°C by using thermostat bath. The viscometer should be calibrated before use to measure the viscosity of the lipid liquid nanoformulation to establish reproducibility [151].

*(9) In Vitro Evaluation*. *In vitro* studies lipid liquid formulation can be performed by using the lipid digestive model. The performance of lipid component during formulation development is evaluated and *in vivo* performance is envisaged. It is essential to develop an *in vitro* dissolution testing performance, i.e., simulated lipolysis release testing [149]. The principle on which it works persistently as a constant pH through the reaction consumes or releases H+ ion. The model consists of the thermostat vessel 37 ± 10°C, which comprises a model GI fluid, and contains digestion buffers, phospholipids, and bile salts. To initiate the process, pancreatic lipase and co-lipase were also added. *In vivo* evaluation: to evaluate the impact of excipients on bioavailability and drug pharmacokinetic profile, *in vivo* studies are performed *via* designing. A comprehensive study of absorption *via* intestinal lymphatic is required, as LBDDS improves bioavailability by enlightening drug intestinal uptake [152, 153].

## 4. Application of LBDDS for Poor Candidates of Antihypertensive Agents

The suboptimal bioavailability of certain antihypertensive agents poses a significant impediment to achieve therapeutic efficacy. By encapsulating poorly soluble antihypertensive agents in lipid carriers, LBDDS offers a mechanism to enhance solubility, protect against degradation, and improve drug absorption, thereby addressing the bioavailability concerns outlined earlier in Table 1. The clinical implications of poor bioavailability in antihypertensive therapy. Reduced efficacy, increased dosage requirements, and compromised patient adherence due to frequent dosing can lead to inadequate blood pressure control, thereby escalating the risk of cardiovascular events.

In previous spans, there are several antihypertensive agents and are encapsulated into the lipid carrier to improve their poor oral bioavailability via protecting the drug from chemical degradation at stomach acidic pH like candesartan cilexitil, protect against enzymatic degradation as liver esterase, and cytochrome P450 causing degradation of most of the antihypertensive drugs, by passing the drug from liver metabolism and so on. LBDDS plays a significant role to conquer these limitations and reduces these difficulties of traditional healing as low solubility, stability, dissolution rate, penetrability, and high hepatic metabolism contribute to produce a highly stable formulation and encapsulate hydrophilic and lipophilic drugs. LBDDS releases the drugs in targeted and controlled manner to decrease the adverse and side effects and produce a tedious delivery system by incorporating the drug into lipid/oil. LBDDS encapsulates the drug and avoids all these constraints. Figure 5 gives an overview of currently used LBDDS for the treatment of hypertension, and various lipid carriers were used from natural and synthetic source for oral delivery of antihypertensive drugs falling in numerous classes as shown in Table 5. The excipients selected for the development of these delivery system must be nontoxic and biodegradable [22, 24, 75, 76, 148, 154–180], [181–210], [211–225], [226–241]. Final oral dosage forms contain functional pharmaceutical excipients that ensure optimal drug performance, facilitate practical and affordable manufacturing, and offer patient-friendly administration [242]. A discussion of real-world cases and studies further substantiates the impact of bioavailability challenges on treatment outcomes.

### 4.1. Limitations of LBBDS

Although lipid-based drug delivery systems can significantly increase the bioavailability of medications that are poorly soluble in water but are not without limitations, the small amount of drug payload that these devices are capable of handling is one major limitation. The lipid composition limits the quantity of medicine that these formulations can efficiently transport and attempts to load larger amounts may result in problems such as phase separation or precipitation, endangering the stability of the formulation. Another challenge is stability; with time, drugs may become less stable due to lipids' susceptibility to hydrolysis and oxidative destruction. The integrity of the formulation and perhaps the therapeutic efficacy of the medication may be jeopardised by the sensitivity to degradation processes. Furthermore, biocompatibility issues surface, especially when certain lipids or surfactants are used at greater concentrations that may irritate or be harmful. Complicacy is increased by the varying absorption of lipid-based formulations in the gastrointestinal system, which is impacted by interindividual physiological variability, bile, and the presence of meals. Specific management guidelines, including taking the medication with certain meals or keeping formulations in a certain way, may make patients less compliant. The shift from laboratory-scale production to large-scale manufacturing is hampered by scale-up problems, which arise from the increasing complexity of maintaining consistent product quality and stability. Lipid-based systems' application is further restricted in the case of hydrophilic medicines, which might not integrate well into lipid matrix. The scene is further complicated by regulatory obstacles, which call for a rigorous and complex approval process due to the heterogeneity in lipid formulations. Final oral dosage forms contain functional pharmaceutical excipients to guarantee best possible drug performance, enable practical and affordable manufacturing, and offer patient-friendly administration. The implementation of LBDDS in antihypertensive therapies, while promising, is not without its challenges. One notable limitation revolves around manufacturability and stability. Mishra et al. (2018) highlighted concerns in oxidative stability, particularly in the context of LBDDS [243]. The susceptibility to oxidative processes poses a significant hurdle in ensuring the consistent quality of these formulations. The disruptive impact of LBDDS on the gastrointestinal (GI) microenvironment raises concerns regarding tolerability and inflammation. The intricate balance within the GI tract is crucial for drug absorption and systemic effects. LBDDS, in certain instances, may perturb this balance, potentially leading to adverse effects. The microenvironmental changes induced by LBDDS warrant careful consideration to mitigate any unintended consequences. These limitations underscore the importance of continued research to address challenges in the manufacturability, stability, and impact on the GI [244]. Strategies to enhance oxidative stability and minimize disruptive effects will be pivotal in advancing LBDDS for optimal application in antihypertensive therapies. While the potential of LBDDS for antihypertensive agents is evident, several challenges merit in-depth consideration. One critical limitation lies in the realm of bioavailability. Despite strides made in improving drug solubility and absorption with LBDDS, variations in patient responses may impact the consistent delivery of antihypertensive agents. Achieving uniform bioavailability poses a persistent challenge, requiring nuanced formulations and optimization.

Patient adherence is another noteworthy concern. LBDDS often involves complex formulations, potentially affecting the ease of administration and patient compliance. Ensuring that these lipid-based formulations are user-friendly and seamlessly integrate into patients' daily routines is essential for the long-term success of antihypertensive therapies. Manufacturing processes further contribute to the challenges associated with LBDDS. The need for sophisticated techniques to achieve reproducibility and scale-up production adds a layer of complexity. Addressing issues related to manufacturing feasibility, scalability, and cost-effectiveness is crucial for the widespread adoption of LBDDS in the pharmaceutical industry. In addition, the impact of LBDDS on the gastrointestinal (GI) microenvironment is a multifaceted concern. Beyond inducing inflammation and affecting tolerability, alterations in the gut microbiota composition may have implications for overall health. The delicate balance in the GI tract is essential for maintaining homeostasis, and any disruption may compromise the therapeutic benefits of antihypertensive drugs delivered through LBDDS. In light of these challenges, ongoing research endeavors must focus on refining LBDDS formulations, streamlining manufacturing processes, and conducting thorough assessments of their impact on patient adherence and GI microenvironment. By addressing these limitations, LBDDS can be harnessed more effectively for the improved delivery of antihypertensive drugs.

LBDDSs present a promising avenue for antihypertensive drug delivery, and certain limitations necessitate a comprehensive examination. Regulatory considerations play a pivotal role, as the introduction of novel lipid formulations demands rigorous approval processes. Navigating regulatory pathways poses challenges that require careful attention, ensuring the safety and efficacy of LBDDS in antihypertensive drug delivery. Such findings are anticipated to reveal important therapeutic implications for the use of oral delivery systems, where microbiome influences might significantly affect medication effectiveness and safety profiles. Despite these limitations, ongoing research endeavors aim to refine lipid-based drug delivery systems, addressing these challenges to unlock their full potential in the realm of pharmaceuticals.

## 5. Conclusion and Future Prospects

LBDDS as a new category of science delivers a bright future prospect and hope for research scientist to achieve a goal for overcoming all the difficulties associated with antihypertensive drugs with poor bioavailability and side effect and provide a vast array to formulations construction with hydrophobic drugs beside through formulation physiologically well accepted class. For the development of these systems, proper understanding of physicochemical properties of drug compounds and gastrointestinal digestion as well as lipid carrier properties and its nature is required. However, it is still necessary to expand the facts and increases the application. More consideration should be given to the properties and characteristics of LBDDS, so that guidelines and candidates identification can be established at the very phase. Proper evaluation and characterization of these systems, their categorization, stability, and regulatory issue subsequently affect this delivery system.

The priority of future research should conduct the bioavailability studies in humans and confirm more specifically the mode of action of the intent and different lipid formulation. On the way of conclusion, LBDDS has a great potential in the treatment of hypertension and effective delivery system for different groups of antihypertensive drugs being developed by choosing a suitable lipid carrier, surfactant, bio enhancers, and so on, so the prospect of these systems looks promising for better management of hypertension with minimum dose to minimal or no adverse effects of antihypertensive agents.

## Figures and Tables

**Table 1:** Antihypertensive agents having poor bioavailability (<50%).

S. no.	Drugs	Therapeutic dose (mg)	Bioavailability (%)	Frequency of administration (times/day)	Drug metabolism	Route	Half-life (T1/2) (hrs)	Adverse effect	Ref. no.
1	Felodipine	2.5–10	15	1	Hepatic	Oral	25	Headache, flush, dizziness, and so on	[20]
2	Isradipine	2.5–10	15–24	1	Hepatic	Oral	8	Headache, dizziness, and edema	[21, 22]
3	Lercanidipine	10–20	10	1	Cyp3a4 hepatic	Oral	8–10	Headache, tachycardia, palpitation, flushing, edema, and so on	[23]
4	Nifedipine	30–120	45–68	1-2	Cyp3a4 hepatic	Oral	2	Headache, flushing, constipation, tiredness, swollen ankle, and so on	[24]
5	Nicardipine	60–120	35	1-2	Liver	Oral	8.6	Headache, flush, dizziness, muscle cramps, tiredness, and so on	[25]
6	Nimodipine	30	13	1	Hepatic	Oral	7–9	Fainting, serve abdominal pain, stomach bloating, and so on	[26]
7	Nisoldipine	10–40	5	1-2	Cytp450 liver	Oral	7–12	Headache, dizziness, pharyngitis, peripheral edema and so on	[27]
8	Verapamil	120–480	20–30	1-2	Liver	Oral	2.8–7.4	Dizziness, bradycardia, constipation, stomach upset, and so on	[28]
9	Diltiazem	180–480	40	1-2	Hepatic	Oral	3–4.5	Swollen head, ankles, feet, headache, dizziness, tiredness, and so on	[29]
10	Lisinopril	10–40	Approx 25	1	None	Oral	12	Cough, headache, vomiting, diarrhea, itching, and blurred vision	[30]
11	Perindopril	4–16	24	1	Hepatic	Oral	1–17	Cough, headache, vomiting, diarrhea, itching, and blurred vision	[31]
12	Benazepril	20–40	37	1	Liver	Oral	10-11	Chest pain, tiredness, weakness, itching, bloating and swelling of the face, and so on	[32]
13	Ramipril	2.5–20	28	1	Hepatic	Oral	2–4	Cough, headache, vomiting, diarrhea, itching, blurred vision, and so on	[33]
14	Enalapril	5–40	40–60	1-2	None	Oral	11–14	Cough, headache, vomiting, diarrhea, itching, and blurred vision	[34]
15	Trandolapril	1–4	40–60	1	Hepatic	Oral	0.7	Appetite lose, nausea, swelling of face, fingers or lower legs, tiredness, and weakness	[35]
16	Fosinopril	10–40	36	1	Liver	Oral	12	Cough, stuffy and runny headache, vomiting, nausea, diarrhea, and itching	[36]
17	Candesartan	8–32	15	1	Intestine wall & hepatic	Oral	9	Sore throat, cough, back, stomach and joint pain, diarrhea, headache, and so on	[37]
18	Eprosartan	400–800	13.10	1	Intestine wall & hepatic	Oral	5–9	Stuffy and runny nose, sore throat, diarrhea, joint and stomach pain, tiredness, and so on	[38]
19	Losartan	50–100	25–35	1-2	Hepatic	Oral	1.5–2.5	Difficulty breathing, blurred vision, tachycardia, numbness in hands, lips and feet, weakness, and heaviness of the legs	[39]
20	Olmesartan	2.5–160	26	1	Hepatic	Oral	15	UTI, swollen ankles, legs, or feet, headache, dizziness, flu like symptoms, nausea, indigestion, and so on	[40]
21	Valsartan	80–320	25	1	Nil	Oral	6	Dizziness, diarrhea, hypotension, back and joint pain, tiredness, bradycardia, and so on	[41]
22	Atenolol	25–100	50	1-2	Liver	Oral	6–9	Hypotension, bradycardia, dizziness, depression, nausea, and so on	[42]
23	Acebutanol	20–1200	40	1	Hepatic	Oral	3-4	Bradycardia, tiredness, dizziness, headache, indigestion, muscle pain, and so on	[43]
24	Nadolol	20–240	30	1	Hepatic	Oral	14–24	Chest pain, blurred vision, fatigue, breathing irregularity, headache, paleness, and so on	[44]
25	Nebivolol	2.5–10	12–98	1	Hepatic	Oral	11–40	Chest pain, headache, dizziness, bradycardia, tingling in feet and hands, weight gain or loss, and so on	[45]
26	Propranolol	40–240	26	2-3	Hepatic	Oral	2–4	Tiredness, dizziness, bradycardia, colds toes and fingers, sickness, nightmares, and so on	[46]
27	Carvedilol	12.5–50	25–35	1-2	Liver	Oral	7–10	Headache, tiredness, weakness, dizziness, nausea and vomiting, and so on	[47, 48]
28	Labetalol	200–1200	25	1-2	Hepatic	Oral	1.7–6.1	Tingling skin, scalp, lightheadedness, tiredness, indigestion, stuffy nose, and so on	[49]
29	Hydralazine	25–50	26–50	1–3	Hepatic	Oral	2–8	Anorexia, headache, nausea and vomiting, appetite loss, diarrhea, dizziness, and so on	[50]
30	Prazosin	0.5–20	55–82	2-3	Liver	Oral	1–3	Drowsiness, dizziness, headache, weakness, palpitations and nausea, and so on	[51]
31	Doxazosin	0.5–16	62–69	1	Hepatic	Oral	16–22	Vertigo, headache, swollen ankles, feet, fingers, abdominal pain, and so on	[52]
32	Furosemide	20–480	60–65	2-3	Hepatic	Oral	4	Nausea and vomiting, constipation and stomach cramping, vertigo, blurred vision, headache, and so on	[53]
33	Amiloride	10–40	15–20	1	Hepatic	Oral	6–9	Headache, nausea and vomiting, appetite loss, abdominal pain, dizziness, and diarrhea	[54]
34	Methyldopa	500–2250	25	2-3	Liver	Oral	1.5–2	Drowsiness, headache, weakness, dizziness, fainting, and nausea and vomiting	[55]

**Table 2:** Different types of nanoformulation used for oral bioavailability enhancement and their advantages and application in biomedical field.

S. no.	Types of nanoformulation	Description	Advantages	Disadvantages	Applications
1	Nanocrystals	These are crystalline particles constructed by various methods such as homogenization, precipitation, milling, and spray drying [77]	(i) Increases dissolution rate via improving aqueous solubility of drug because bioavailability is enhanced		(i) It is helpful for controlling the level of cholesterol and triglycerides [78]
			(ii) Composition is safe		
			(iii) Appropriate for parenteral route [77]		(ii) Used in hyperthermic chemotherapy and in cancer treatment [79, 80]

2	Polymeric nanoparticles	These are prepared as nanocapsules and nanospheres by using different techniques such as ionic gelation, emulsification, and nanoprecipitation [81]	(i) Site-specific targeting		(i) Targeting drug and gene delivery system [82, 83]
			(ii) Toxicity reduction		
			(iii) Drug release in controlled manner		
			(iv) Increased permeability		(ii) Engineering of tissue [84]
			(v) Drug protection from chemical and enzymatic degradation [81]		

3	Liposomes	Liposomes are synthetic vesicles prepared by using lipid bilayers; these vesicles are further subdivided into two groups unilamellar and multilamellar, which are able to dissolve to both water or lipid soluble drugs at the same time [85, 86]	(i) Drug stability is increased	(i) Production cost is high	(i) Used for delivering various drugs or biomolecules such as protein/peptide [88], hormones [89], enzymes [90], anticancer drugs [91], and so on
			(ii) Degradable and biocompatible	(ii) Drug leakage	
			(iii) Enhance the efficacy and toxicity is reduced	(iii) Poor solubility	
			(iv) Increased penetrability reduces side effects [87]	(iv) Inadequate stability [87]	

4	Micelles	Micelles are spherical amphiphilic copolymers nanosized particles prepared by supramolecular assemblage and have a shell assembly with lipidic interior detached from the hydrophilic outward [92]	(i) Greater drug loading capacity		(i) Best carrier system for water insoluble and lipophilic drug molecules [93], e.g., paclitaxel [94], doxorubicin [95], and so on
			(ii) Better stability		
			(iii) Extended circulation period		
			(iv) Lesser side effects		
			(v) Protect against degradation [92]		

5	Dendrimers	These are nanosized symmetrically balanced molecules where a small atom or groups of atoms are enclosed in symmetric branches like a tree acknowledged as dendrons [96, 97]	(i) Easy functionality and uniformity		Medical application: (i) Used as an analog of protein and enzyme [98] (ii) Mimic the variety of biomolecule [99]
			(ii) Protect against enzymatic degradation		
			(iii) Molecular size and weight can be controlled		
			(iv) Biocompatible [86]		To enhance the solubility and stability of drug molecules [100]
					To achieve the targeted site-specific drug delivery

6	Nanosuspension	These are the colloidal dispersion of nanosized bioactive or drug molecules stabilized by the surfactants formulated via bottom-up and top-down techniques [101]	(i) Increased the dissolution rate via solubility and bioavailability of drug molecules		(i) Produce the sustained release of drug over 24 h, e.g., ketoprofen [103]
			(ii) Suitable for water-soluble drug molecules		(ii) Administered parenteral routes such as intra-articular and intraperitoneal and increased the drug efficacy [102, 103]
			(iii) Reduces the dose size		
			(iv) Improves physiochemical properties of drugs		
			(v) Achieve passively drug targeting [102]		
7	Carbon nanotubes	These tubes are formed by graphene as carbon hexagonal matrix as seamless hollow cylindrical fibers, which are rolled along separately at specific chiral angles. The diameter is around 0.5–50 nm [86, 104, 105]	(i) Small size and light in weight		(i) Used as a fillers
			(ii) Mechanical strength is high		(ii) Used in gene and cancer treatment
			(iii) Have a larger surface area [84, 103]		(iii) They are used to increase the solubility and stability of drug candidates [104–106]

8	Nanogels	These are 3D hygrogels composition in nanosized range prepared by swellable crosslinked polymers having capacity to hold water [107]	(i) Are fabricated in different shapes		(i) Used to protect drug candidate and targeted drug delivery
			(ii) Crosslinked structural integrity		(ii) Drug release in controlled manner [110, 111]
			(iii) Highly biocompatible		(iii) In the central nervous system, antiviral therapy of HIV-1 infection [112]
			(iv) High loading capacity [108, 109]		

9	Lipid-based drug delivery system	LBDDS for orally administered entities/drugs commonly comprises of a liquefied mixture of two or more excipients including triglycerides lipids/oils, fractional glycerides, and natural lipids , surfactants, and cosurfactants	(i) Controlled and targeted drug release		(i) To improve the stability in GIT, increases solubility bioavailability of poorly aqueous soluble or insoluble drugs
			(ii) Pharmaceutical drug stability and compatibility		(ii) Can be used for the delivery of different types of new drug chemical entity having very poor bioavailability, protein, peptides, nucleic acid, and so on [113–115]
			(iii) Improved drug solubility and bioavailability		(iii) Formulation approaches include nano/microemulsion, SNEDDS,, SMEDDS, liposomes, and so on [4]
			(iv) Lipophilic and hydrophilic drug encapsulation is feasible		
			(v) Biodegradable		
			(vi) Formulation flexibility		
			(vii) Poor uncertainty profile		
			(viii) Inert, noninvasive development of the vesicular system which is presented for immediate commercialization [4]		

**Table 3:** Classification of LBDDS their characteristics, advantages, and disadvantages [123, 124].

Formulation class	Class 1	Class II	Class IIIA (fine emulsion)	Class IIIB (microemulsion)	Class IV
Materials used in formulation	Oils without surfactants	Oils and O/W soluble surfactants	Lipid/oils, surfactants-cosurfactant ratio, cosolvents (both aqueous soluble and insoluble components)	Lipid/oils, surfactants-cosurfactant ratio, cosolvents (both aqueous soluble and insoluble components)	Water-soluble surfactants and cosolvents (no oils)
Characteristics	Nondispersible	Emulsification (SEDDS) with aqueous-insoluble substances	SEDDS/SNEDDS/SMEDDS molded using aqueous solvable substances	SEDDS/SMEDDS formed using aqueous soluble substances and the amount of oil is less	Disperses classically to produce a micelles solution
Digestion characteristic	Requires digestion	Ingested easily	Ingestion not essential	Ingestion not compulsory	Partial ingestion
Advantages	Simple GRAS capsule compatibility	Unlikely, on dispersion loose solvent capacity	On dispersion, clear or almost clear dispersion	On dispersion, clear or almost clear dispersion	(i) Formulation has good solvent capability
Disadvantages	Poor solvent capability	Relatively coarse O/W dispersion, ingestion probably but not decisive	On dispersion, possibility is loose of solvent capability or ingestion	On dispersion, possibility is loose of solvent capability or ingestion	On dispersion, possibility is loose of solvent capability

**Table 4:** Commonly used oil, emulsifiers, coemulsifiers, and cosolvents in LBDDS.

Brand name	Chemical definition	HLB value	Suppliers/manufacturer	Excipients
Tween 20 (T20)	PEG-20 sorbitan monolaurate	16.7	Atlas/ICI	Emulsifier
Tween 60 (T60)	Polysorbate 60	14	Atlas/ICI	Emulsifier
Tween 65 (T65)	PEG-20 sorbitan tristearate	11	Atlas/ICI	Emulsifier
Tween 80 (T80)	PEG-20 sorbitan monooleate	15	Atlas/ICI	Coemulsifier
Span 20 (S20)	Sorbitan monolaurate	8.6	Atlas/ICI	Emulsifier
Span 60 (S60)	Sorbitan monostearate	4.7	Atlas/ICI	Coemulsifier
Span80 (S80)	Sorbitan monooleate	4.3	Atlas/ICI	Coemulsifier
Brij-30	PEG-4 lauryl ether	9.7	Atlas/ICI	Emulsifier
Arlasolve DMI	Di-methyl isosorbide	—	Atlas/ICI	Coemulsifier
Capmul MCM-C8	Glyceryl caprylate	5-6	ABITEC	Emulsifier
Lecithin	L-a-Phosphatidylcholine	4–9	Alfa Aesar	Emulsifier
Cerex ELS 250	PEG-25 hydrogenated castor oil	11	Auschem SpA	Emulsifier
Akoline MCM	Caprylic/capric glycerides	5-6	Aarhuskarlshamn	Coemulsifier
Cremophor EL Cremophor ELP	PEG-35 castor oil	12–14	BASF	Emulsifier
Cremophor RH40	PEG-35 hydrogenated castor oil	13	BASF	Emulsifier
Pluronic L44	Block copolymer of ethylene oxide and propylene oxide	12–18	BASF	Coemulsifier
Lutrol F 68	Polaxomer 188	29	BASF	Coemulsifier
Cremophor RH40	Polyoxyl-40-hydrogenated castor oil	13	BASF	Emulsifier
SLS	Sodium lauryl sulfate	40	Canadian Alcolac	Coemulsifier
Carbitol	Diethylene glycol monoethyl ether	—	Dow Chemicals	Coemulsifier
TPGS	Tocophersolan, D-*α*-tocopheryl PEG-1000 succinate	13	Eastman	Emulsifier
Labrafil M 2125 CS	PEG-6 corn oil	4	Gattefosse	Emulsifier
Labrafil M1944CS	PEG-6 apricot kernel oil	4	Gattefosse	Emulsifier
Labrasol	PEG-8 caprylic/capric glycerides	14	Gattefosse	Emulsifier
Labrafac CM 10	PEG-8 caprylic/capric glycerides	>10	Gattefosse	Emulsifier
Labrafil WL 2609 BS	PEG-8 corn oil	6-7	Gattefosse	Emulsifier
Peceol	Glyceryl monooleate	3-4	Gattefosse	Emulsifier
Plurol oleique CC 497	Polyglyceryl-6 dioleate	6	Gattefosse	Coemulsifier
Caprol® 6G20			Abitec CoCalgene	
Hodag PGO-62				
Lauroglycol 90	Propylene glycol monolaurate	5	Gattefosse	Coemulsifier
Lauroglycol FCC	Propylene glycol monolaurate	4	Gattefosse	Coemulsifier
Transcutol P	Diethylene glycol mono ethyl ether	—	Gattefosse	Coemulsifier
Labrafil 1944	PEG-6 apricot kernel oil	4	Gattefosse	Coemulsifier
HCO-40	Polyoxyethylene hydrogenated castor oil 40	13	Nikkol	Emulsifier
HCO-60	PEG-60 hydrogenated castor oil	14	Nikkol	Coemulsifier
Emulphor El-620	Ethoxylated castor oil	12–15	Rhodia	Emulsifier

**Table 5:** Various LBDDSs of antihypertensive compounds.

S. no.	Drug candidate	BCS class	Absorbent/polymers/bioenhancer/other excipients	Lipid/oils	Surfactant	Cosurfactant/other excipients	Formulation approach	Year of publication	Reference no.	Outcomes
*Calcium channel blockers*										
1	Amlodipine	Class I		Oleic acid	Tween 80	Transcutol P	NE	2009	[154]	This study signifies that nanoemulsion serves as a potential vehicle for improving the transdermal delivery of amlodipine
2	Amlodipine basilate	Class 1	—	Labrafil M	Tween 80	Ethanol	NE	2011	[155]	This study was indicating 3-fold increase in the whole residence time of NE that is suggested NE as drug carriers for improving the bioavailability in comparison of marketed formulation
3	Felodipine	Class II	Aerosil 200	Acconon E	Cremophor EL	Lutrol E300	SNEDDS	2013	[20]	Felodipine containing SNEDDS and S-SNEDDS has significant potential to improve its absorption through git and may serve a capable delivery *via* oral administration
4	Isradipine	Class II		Vitamin E TPGS	Sodium lauryl sulfate		Nanosuspension	2012	[156]	*In vitro* dissolution study showed that the dissolution rate of nanosuspensions (98.60%) and saturation solubility (98.76 *μ*g/ml) compared with the coarse drug (11.53% and 14.1 *μ*g/ml, respectively) had been significantly enhancing and pharmacokinetic study showed increases in AUC_0–48_ by 2.0-fold and increases the *C*_max_ and *T*_max_ in comparison to pure drug suspension
5	Isradipine	Class II	Neusilin us2	Labrafil® M2125	Capmul® MCM L8	Cremophor® EL	S-SEDDS	2014	[22]	The results of this study showed that *in vitro* drug release rate was increased about 18-fold and *in vivo* bioavailability was increased about 2.5-fold from marketed formulation
6	Isradipine	Class II	—	Trimyristin and tristerin	Poloxamer 188		SLN	2014	[20]	The drug release from SLNs formulation is found to be around 99% within 12 hours
7	Isradipine	Class II	—	Steric acid, glyceryl monostearate	—	—	SLN	2016	[157]	This study included SLN prepared with two lipids in different concentrations. The pharmacodynamics studies of optimized formulation showed reduction in BP upto 36 hrs and confirmed the suitable carrier for oral administration
8	Isradipine	Class II	—	Glyceryl monostearate: soya lecithin	—	Eudragit L100, rutin	SLN	2018	[158]	Pharmacokinetic study shows the 3.2–4.7-fold increase in the bioavailability of coated SLN of isradipine as compared to conventional drug suspension. *In vivo* studies show greater absorption orally
9	Isradipine	Class II	—	Tricetin	Tween 20	Transcutol	NE	2020	[159]	This study showed greater dissolution profile and solubility of isradipine
10	Lercanidipine	Class II	Neusilin	Capmul MCM L8	Tween 80	PEG 400	S-SEDDS	2012	[160]	Optimized S-SEDDS showed the greater dissolution rate as compared to pure drugs and proved that SEDDS formulations are alternative approaches for oral route administration
11	Lercardipine HCL	Class II	Avicel PH 101	Peppermint oil	Propylene glycol	PEG 400	S-SEDDS	2020	[24]	Optimized formulation showed enhanced solubility and dissolution profile of the lercardipine hydrochloride in comparison with pure drug
										The S-SEDDS had shown good stability
12	Nifedipine	Class II	—	Cremophor®E L (polyoxyl 35 castor oil)	Caprylic/capric glyceride	Transcutol® HP (diethylene glycolmonoethy l ether)	SNEDDS	2014	[161]	Optimized formulation shows significant increase in the AUC _0–12 h_ and 2.9 folds in comparison to pure drug powder and the AUC_0–12 h_ of nifedidine loaded SNEDDS and drug powder was 4082.6 ± 621.7 and 1413.4 ± 388.4 ng/mL·h, respectively
13	Nifedipine	Class II	Aerosil® 200	Imwitor® 742	Cremophor (®) RH40	Span (®) 80	SNEDDS	2014	[162]	It is concluded that nifedipine loaded SNEDDS is favorable dosage form with good *in vitro* studies
14	Nimodipine	Class II	—	Glucire 44/14	Transcutol p	Plurol oleique CC 497	SEDDS	2008	[163]	SEDDS formulation shows significant improvement in the in vitro and in vivo performance, i.e., it is a novel effective alternative for the development of nimodipine formulation
15	Nimodipine	Class II	—	Peppermint oil	Cremphor EL	PEG 400	SEDDS	2018	[164]	Study successfully discriminated the influence of design variables on %age of the nimodipine in aqueous which signifies the segment of SEDDS arranged for instant absorption
16	Nimodipine	Class II	—	Peceol	Transcutol P	PEG 400	SNEDDS	2019	[165]	Optimized SNEDDS formulation of nimodipine shows improved *in vitro* dissolutions and absorption profile of the drugs and also indicate that the stability of drug in formulation is good
17	Nitrendipine	Class II	—	Tripalmitin/glyceryl monostearate/cetyl palmitate	Soy phosphatidylcholine 95%	Poloxamer 188	SLN	2006	[166]	Bioavailability of lipophilic drugs like nitrendipine could be improved by suitably incorporating into nimodipine SLN and enhancing the bioavailability of the drugs from 3.21 to 5.35 folds on i.d. administration
18	Nitrendipine	Class II	Carbopol	Trimyristin	Tween 80	—	SLN and NLC hydrogels	2008	[167]	These transdermal gels show abundant efficacy and a feasible option of effective and controlled management of high blood pressure
19	Nitrendipine	Class II	—	Caproyl 90®	Tween 80	Transcutol P®/solutol HS-15®	Intranasal NE	2009	[168]	*In vivo* absorption studies show improved absorption and an instant onset of action and relative bioavailability of 60.44%, that is greater than the marketed tablets and pure suspension
20	Niterndipine	Class II	—	Capmul MCM: triacetin	Kolliphor ELP	Transcutol HP	NE gel	2020	[169]	This gel improves the poor penetration and it could be used as a potential carrier for the delivery of nitrendipine

*ACE inhibitors*										
21	Captopril	Class I	Curcumin	Glyceryl monooleate	Tween 20	PEG 400	NE	2015	[170]	This study shows that curcumin synergism effect on captopril activity and NE formulation increase the poor solubility of the drug
22	Captopril	Class I	—	Castor oil	Kolliphor RH40: Kolliphor EL	Glycerol	SEDDS	2020	[171]	This study provides indication for a sustained release from SEDDS formulation of captopril drug
23	Ramipril	Class II	—	Safsol 218	Tween 80	Carbitol 18	NE	2007	[172]	This study revealed that optimized formulation of ramipril NE could be used for geriatric and pediatric patients in liquid unit dosage form. *In vitro* release was highly significant as compared with pure suspension and marketed formulation
24	Ramipril	Class II	—	Safsol 218	Cremophor EL	Carbitol	NE	2008	[173]	Bioavailability of ramipril is increased 4.29-fold to drug suspension and 1.76-fold increase that of marketed tablet. Degradation rate was slow in NE with aqueous phase (buffered solution pH-5.0) in comparison to other formulation, i.e., these results indicated improved stability of ramipril in NE
25	Ramipril	Class II	—	Glyceryl monooleate	Span 20	—	SLN	2011	[174]	The optimized formulation shows the prolonged drug release as compared to other formulations with different lipids and surfactants
26	Ramipril	Class II	—	Capmul PG8 NF	Gelucire 44/14	Transcutol P	SNEDDS	2016	[175]	Optimized NE formulation shows significant improved stability, solubility, and dissolution rate of the ramipril
27	Ramipril	Class II	Syloid	Capmul MCM/Polyoxyethylene hydrogenated castor oil	—	Transcutol P	S-SNEDDS	2019	[176]	Results of this studies show significant improvement in solubility and stability against heat, moisture, and mechanical stress during manufacturing and storage

*AT II receptor blockers*										
28	Candesartan cilexetil	Class II	—	Miglyol 812	Labrasol	Tween 80/Cremophor EL	SMEDDS	2010	[177]	The optimized formulation shows the higher drug release rate than the marketed formulation; this study indicates the SMEDDS had a potential to enhance the solubility and dissolution rate of the poorly soluble drug compound
29	Candesartan cilexetil	Class II	—	Lauroglycol 90	Tween 40	Transcutol P	SNEDDS	2015	[178]	This study shows significant improvement, permeability, and oral bioavailability of the drugs from the SNEDDS formulation
30	Candesartan cilexetil	Class II	—	Peppermint oil	Cremophor RH40	Labrasol	SNEDDS	2019	[179]	In this study, SNEDDS formulation of drugs shows rapid onset of action and prolonged therapeutic activity
										SNEDDS improved the oral bioavailability of candesartan about 1.69-fold as compared with the marketed formulation
31	Candesartan cilexetil	Class II	Aerosil and Avicel 101	Cinnamon oil	Tween 80: polaxomer 407	Transcutol P	S-SNEDDS	2017	[180]	This study concluded that S-SNEDDS is a favorable approach to enhance the wettability, poor solubility, dissolution rate, and stability of candesartan cilexetil
32	Candesartan cilexetil	Class II	—	Capryol 90®	Tween 80	Transcutol P	SNEDDS	2019	[181]	The optimized formulation of SNEDDS shows promising delivery system with rapid onset of action and prolonged therapeutic effect of drugs
33	Candesartan	Class II	—	Capryol 90®	Captex 500	Labrasol	SNEDDS	2018	[182]	This study shows the higher drug release from the formulation into the blood stream as compared to marketed formulation and pure suspension
34	Candesartan cilexetil	Class II	—	Triacetin oil	Cremophore RH 40	Transcutol P	SNEDDS	2020	[183]	These studies indicate the increased dissolution profile of candesartan cilexitil (98%) in comparison to pure drug suspension (45%)
35	Candesartan cilexetil	Class II	—	Soybean oil	Solutol HS-15	Tween 80	NE	2011	[184]	The results of this studies show that NEs are very effective formulation approach for improving the oral absorption of insoluble drug candidate. 8 candesartan enhanced 10 folds AUC_0–*t*_ results when incorporated into the NE
36	Candesartan cilexetil	Class II	—	Soybean lecithin: glycerol monostearate	Tween 80	—	SLN	2012	[185]	The pharmacokinetic studies show improvement in oral bioavailability over 12-fold after encapsulation into SLN
37	Candesartan cilexetil	Class II	Dynasan 116	Egg-lecithin (E-80)	Poloxamer-188	—	SLN	2014	[186]	These studies conclude that optimized SLN of drug showed 2.75-fold enhancement in the oral bioavailability and confirmed SLN is suitable for carrying candesartan cilexitil for oral route administration
38	Candesartan cilexetil	Class II	Hydroxypropyl methylcellulose	Pluronic® F 127		—	Nanocrystals	2016	[187]	This study investigated that nanocrystals are suitable for improvement of the oral bioavailability of the candesartan cilexitil
39	Candesartan cilexetil	Class II	—	Pluronic P85	Span 60	—	Niosomes	2014	[188]	This study concluded that niosomes increase the solubility and stability of drugs against the bile distruption and had an great potential to increase the oral bioavailability of poorly water-soluble drug candidates via encapsulation in lipid carrier
40	Candesartan cilexetil	Class II	Maltodextrin/diacetyl phosphate	Cholesterol/	Span 60	—	Proniosomes	2016	[189]	These studies indicate proniosomes of candesartan a promising formulation for the enhancement of oral bioavailability and patient compliance
41	Candesartan cilexetil	Class II	Chitosan	Peceol™	Span 60	—	Niosomes	2021	[190]	These studies conclude that chitosan coated or uncoated niosomes of candesartan cilexitil shows better oral absolute bioavailability
42	Olmesartan midoxomil	Class II	—	Capryol 90	Tween 20	Tetraglycol	SMEDDS	2009	[191]	This study shows that SMEDDS increases the relative oral bioavailability about 170% as compared to that pure drug suspension. SMEDDS increased the solubility and permeability by inhibiting the efflux pump
43	Olmesartan midoxomil	Class II	—	Capryol 90	Labrasol	Transcutol	SMEDDS	2011	[192]	This study concluded that SMEDDS enhances the oral bioavailability of olmesartan medoxomil *in vivo* 2.7-fold as compared to pure drug suspension
44	Olmesartan midoxomil	Class II	—	Acrysol EL 135	Tween 80	Transcutol P	SMEDDS	2013	[193]	This study concluded *in vivo* and *ex vivo* diffusion rates from SMEDDS and is greater than the pure drug suspension of the olmesartan medoxomil. So, it is a promising delivery system for improving the oral bioavailability of poorly water-soluble drug candidates
45	Olmesartan midoxomil	Class II	—	Oleic acid	Tween 80	Transcutol	SMEDDS	2019	[194]	This study showed SMEDDS increased the solubility and bioavailability of hydrophobic olmesartan medoxomil while reducing the side effects of drug such as enteropathy
46	Olmesartan midoxomil	Class II	—	Soyabean oil 700	Sefsol 218	Solutol HS-15	NE	2014	[75]	The pharmacokinetic studies showed increased AUC about 2.8 folds and sustained release upon oral administration
47	Olmesartan midoxomil	Class II	Aerosil 200	Capryol 90	Cremophor RH40	Transcutol HP	S-SNEDDS	2016	[76]	This study concluded that *in vitro* dissolution rate of olmesartan is increased, and pharmacokinetic behavior of drug is also improved as compared to the pure drug suspension
48	Olmesartan midoxomil	Class II	Silicon dioxide	Capmul MCM®	Tween 80®	PEG 400	S-SEDDS	2012	[195]	This study concluded that S-SEDDS had a better handling potential as compared with NE and *in vitro* release was similar
49	Telmisartan	Class II	—	Acrysol® EL 135	Tween® 20	Carbitol®	SNEDDS	2011	[196]	The *in vivo* study shows 7.5-fold enhancement of the oral bioavailability of telmisartan from SNEDDS as compared to the pure drug suspension
50	Telmisartan	Class II	Neusilin US2	Oleic acid	Tween 80	PEG 400	S-SMEDDS	2012	[197]	This study concluded that telmisartan-loaded S-SMEDDS shows improvement in dissolution profile of the drug as compared with pure drug suspension
51	Telmisartan	Class II	Micro-crystalline cellulose	Castor oil	Tween 20	Propylene glycol	S-SMEDDS	2014	[198]	This study concludes that *in vitro* release of telmisartan from S-SMEDDS was 100% in about 120 min, that was higher than the pure drug suspension
52	Telmisartan	Class II	—	Capmul MCM®	Tween 80	Propylene glycol	SMEDDS	2015	[199]	The result concluded that the pharmacokinetic study shows 1.54-fold increase in bioavailability of telmisartan-loaded SMEDDS in comparison with pure drug and marketed formulation
53	Telmisartan	Class II	—	Labrafil 1944	Kolliphor ELP: span 80	PEG 400: Ethanol	SNEDDS	2017	[200]	This study concluded that telmisartan-loaded SNEDDS could increase the oral bioavailability as compared to pure drug suspension
54	Telmisartan	Class II	Phospholipid	Capryol 90	Tween 80	Tetraglycol	SMEDDS	2018	[201]	The optimized formulation shows pH independent high dissolution of temisdartan. So, this study suggested that the phospholipid complex with SMEDDS is beneficial for enhancement of the dissolution of hydrophobic drug
55	Telmisartan	Class II	—	Cinnamon	Gelucire 44/14	Transcutol	SMEDDS	2018	[202]	The results of the study concluded that in vivo studies of telmisartan-loaded SMEDDS had successfully increased the *C*_max_ and AUC of the drug as compared to pure drug suspension
56	Telmisartan	Class II	—	Capmul MCM EP	Labrasol	Transcutol HP		2019	[203]	The developed and optimized formulation of telmisartan-loaded SMEDDS shows significant improvement in the dissolution rate and profile in comparison to pure drug suspension. This research shows that SMEDDS has a great potential for delivering the BCS-II class drugs
57	Telmisartan	Class II	—	Capmul® MCM	Cremophor® RH40	Tetraglycol	Su-SEDDS	2020	[204]	The *in vivo* pharmacokinetic study shows that oral bioavailability of drug from Su-SEDDS was about 1.2-fold greater than the pure drug suspension
58	Telmisartan	Class II	—	Capmul PG 8	Cremophor RH 40	Transcutol P	S-SMEDDS	2021	[205]	This study shows that the *in vitro* dissolution test is significantly higher than the plain telmisartan
59	Telmisartan	Class II	Carbopol 934	Labrafil®M 2125 CS	Acrysol®EL 135	Carbitol®	NE gel	2015	[206]	The study concluded that optimized formulation of NE gel showed higher bioavailability as compared to conventional gel and it showed greater permeation and penetration rate in *in vivo* and *in vitro*
60	Telmisartan	Class II	—	Oleic acid	Tween 80	PEG	NE	2017	[207]	Optimized formulation showed greater stability and drug release in comparison with conventional formulation and showed enhanced bioavailability
61	Telmisartan	Class II	Chitosan	Sefsol 218 and oleic acid	Tween 20	Transcutol P	Mucoadhesive nanoemulgel	2021	[208]	This study concluded that the telmisartan mucoadhesive nanoemulgel, coated with chitosan, emerged as a promising technique for the treatment of tuberculosis, facilitating direct nose-to-brain delivery based on in *vivo* and *ex vivo* results
62	Telmisartan	Class II	—	Soya PL phospholipids	Brij 35	—	Proniosomes	2020	[209]	The optimized formulation *in vivo* study showed Cmax enhances 1.5-fold and AUC increases about 3-fold and also produces sustained effect when it is compared with the marketed tablets
63	Telmisartan	Class II	—	Cholesterol	Span 60	—	Niosomes	2021	[210]	This study concluded that telmisartan encapsulated niosomes had shown better drug release when compared with pure drug
64	Telmisartan	Class II	—	Soya lecithin	Span 80	Tween 80	Transferosomes	2018	[211]	The results of the optimized formulation showed better entrapment efficacy (50.72%), zeta potential (27.6 mv%), and drug diffusion (−58 ± 2.4%) for effective control of cardiovascular disorders
65	Losartan potassium	Class II	Egg-lecithin	Cholesterol	Span 60	—	Proniosomes	2009	[212]	The results of *in vivo* pharmacokinetic study showed significant increase in the bioavailability about 1.93-fold as compared with oral formulation of losartan potassium
66	Valsartan	Class II	—	Capmul MCM	Tween 80	PEG 400	SMEDDS	2010	[213]	Results of the study showed higher drug release and bioavailability of valsartan from SMEDDS
67	Valsartan	Class II	—	Castor oil	Tween 80	PEG 600	SEDDS	2011	[214]	The optimized formulation shows adequate improvement in the drug release and stability of the drug from valsartan loaded SEDDS
68	Valsartan	Class II	—	Labrasol	Tween 20	PEG 400	SNEDDS	2012	[215]	The optimized formulation showed significantly enhanced solubility and stability of the valsartan loaded in SNEDDS as compared with marketed formulation. This improvement in the solubility could lead to higher drug oral bioavailability
69	Valsartan	Class II	Neusilin US2	Capmul MCM	Kolliphor HS-15	PEG 400	S-SEDDS	2015	[216]	The results of the study showed increase in the bioavailability of valsartan from S-SEDDs about 1.6 folds as compared with plain drug
70	Valsartan	Class II	Florite® PS-10 and Vivapur® 105	Capmul MCM	Tween 80	Transcutol® P and poloxamer 407	S-SuSMEDDS	2017	[217]	The pharmacokinetic study concluded in rats the relative bioavailability of the S-SuSMEDDS granules and DIOVAN powder was about 107% and 222%, respectively. Therefore, this technique has a great potential for developing solid dosage form of liquefied formulation for improving oral bioavailability of hydrophobic drugs
71	Valsartan	Class II	—	Capmul® MCM	Tween® 20	Transcutol® P	SuSMEDDS	2017	[218]	Results showed excellent *in vivo* and *in vitro* bioavailability of the drug from optimized formulation
72	Valsartan	Class II	L-HPC and Florite® PS-10	Capmul MCM	Tween 80	Gelucire® 44/14	S-SuSMED	2019	[219]	The study concluded that optimized S-SuSMED tablets enhanced the oral bioavailability in rat about 177–198% as compared with plain valsartan and DIOVAN
73	Valsartan	Class II	—	Phospholipon 90G	Sodium deoxycholate		Nanotransferosomes	2012	[220]	The i*n vivo* study on Wistar rats showed that nanotransferosomes enhance the transdermal delivery of valsartan drug and produces the prolonged control on blood pressure up to 48 hrs
74	Valsartan	Class II	—	Phospholipon 90G	Span 40	—	Transferosomes	2020	[221]	This study concluded that valsartan-loaded transferosomes patch will be effective in reducing the frequency of dosing, as it has produced sustained effects and enhanced patient compliance
75	Valsartan	Class II	—	Cholesterol	Span 60	—	Niosomes	2019	[222]	The results of this study showed controlled release about 24 hrs (98.55) of the valsartan from the niosomes
76	Valsartan	Class II	Lecithin	Cholesterol	Span 60	—	Proniosomes	2011	[223]	The study concluded that proniosomes prepared with span 60, cholesterol, and lecithin have an high encapsulation efficacy, release rate, and stability as compared with formulation composition

*Beta-blockers*										
77	Atenolol/metaprolol/danazol	Class III,/II/I	—	Lauroglycol ® 90	Pluronic® P104	Pluronic® L62 or L81	Nanoemulsion	2007	[150]	The study concluded that BCS-1 and II drugs show higher permeability as compared with plain drugs. Atenolol increases 2.5 folds, danazol increases 3.2 folds, and metoprolol increases 1.4 folds
78	Carvedilol	Class II	—	Labrafil M 1944CS	Tween 80	Transcutol P	SMEDDS	2005	[224]	The results of the study showed that increase *in vitro* dissolution rate of the carvedilol from SEDDS and SMEDDS about 2 folds than marketed tablets
79	Carvedilol	Class II	—	Gelucire 44/14	Lutrol F68	Transcutol P	Self-emulsifying osmotic pump	2007	[225]	The results of the studies show that drug release from self-emulsifying osmotic pump was controlled and follows zero-order kinetic and improved the oral bioavailability
80	Carvedilol	Class II	—	Capmul PG8	Cremophor EL	Transcutol HP	SNEDDS	2011	[226]	This study shows accelerated stability of the optimized formulation for 6 months. *In situ* perfusion study on Wistar rats showed improved permeability and absorption potential about several folds in comparison to marketed formulation
81	Carvedilol	Class II	—	Capmul MCM	Nikkol HCO 50	—	SNEDDS	2012	[227]	The results of the studies were stable during the 6 months of study periods. And optimized formulation had a potential of enhancement of oral bioavailability of carvedilol
82	Carvedilol	Class II	Hydroxypropyl methylcellulose/polaxomer	Oleic acid	Labrafil	Labrafac PG	SEDDS	2014	[228]	This study concluded that carvedilol loaded SEDDS showed drug permeability through the rat intestine was about 2.76 folds greater than the control
83	Carvedilol	Class II	—	Caproyl 90	Tween 20	Transcutol HP	SEDDS	2017	[229]	The results of this study concluded that optimized formulation of carvedilol loaded SEDDS expressed rapid onset of action and improved the antihypertensive activity
84	Carvedilol	Class II	—	Cremophor RH40	PEG 400	HPMC-E5	Su-SEDDS	2020	[230]	The *in vivo* results of the study showed increase in the permeability and bioavailability about 2.2 folds and 3.2 folds, respectively
85	Carvedilol	Class II	—	Castor oil	Solutol or Kolliphor RH40	Solutol or Kolliphor RH40	L-SEDDS	2021	[231]	The study concluded increase in the *in vitro* dissolution and stability of the drug as compared with the plain drug
86	Carvedilol	Class II	Aerosil	Peceol™	Tween® 80	Labrasol®	S-SNEDDS	2021	[232]	The results of the study showed that increases in the solubility, dissolution rate, and oral bioavailability were about 6.1 folds, 1.8 folds, and 1.4 folds, respectively, of the carvedilol from S-SEDDS formulation
87	Carvedilol	Class II	—	Oleic acid/isopropyl myristate	Tween 80	Transcutol P	Transdermal NE	2008	[233]	The results of the study concluded that there were increases in solubility of the drug in 4500 folds, decrease in the activation energy about 88% during thermodynamic studies, and the optimized formulation was nonirritant for the skin suggested by irritation studies
88	Carvedilol	Class II	—	Steric acid	Polaxomer 188	Sodium taurocholate and ethanol	SLN	2009	[234]	The results of the study showed decrease in the bioavailability of carvedilol on increasing the concentration of polaxomer 188 in formulations about 4.91–2.84 folds after intraduonal administration in Wistar rats
89	Carvedilol	Class II	Neusilin US2	Stearic acid	—	Dichloromethane and methanol	SLN	2010	[235]	This study concluded that SLN increases the efficacy, release rate, and stability of the drug in comparison to plain nanosuspension
90	Carvedilol	Class II	N-Carboxymethyl chitosan	Monoglyceride	Soya lecithin and poloxamer 188	—	SLN	2012	[236]	In vivo study rats showed SLN coated with n-carboxymethyl chitosan increases the bioavailability of carvedilol in comparison to uncoated after oral administration
91	Carvedilol	Class II	—	Compritol or Precirol	Poloxamer 188	Lecithin	Intranasal SLN	2016	[237]	The *in vivo* pharmacokinetic study showed increases in absolute bioavailability (350.63%) in comparison to oral carvedilol formulation (24.11%)
92	Carvedilol	Class II	—	Phospholipon 100 H and cholesterol	Ethanol	Transcutol HP	Transdermal ethosomal gel	2019	[238]	The study concluded by ethosomes having a great potential to increase the skin penetration with extended carvedilol antihypertensive action
93	Carvedilol	Class II	—	Lipid	Ethanol	Propylene glycol	Transdermal ethosomes	2021	[239]	In vitro studies of drug release showed sustained release of the drug from ethosomes and ethosomal gel and enhanced the penetration via skin
94	Propranolol hydrochloride	Class I	—	Lecithin	Sorbitol	—	Proliposomes	1995	[240]	This study concluded that proliposome had a great potential for the sustained effect when applied to mucosal membrane
95	Propranolol hydrochloride	Class I	—	Cholesterol	Phosphatidyl ethanolamine	—	Liposomal gel	2015	[241]	In vivo study showed increases in the propranolol hydrochloride concentration in skin about 74 folds as compared to plain drug

BCS, Biopharmaceutical Classification System; SEDDS, self-emulsifying drug delivery system; SNEDDS, self-nanoemulsifying drug delivery system. SMEDDS, self-microemulsifying drug delivery system; S-SEDDS, solid self-emulsifying drug delivery system; SLN, solid lipid particles; NLC, nanostructured lipid carriers; PEG, polyetylene glycol;. NE, nanoemulsion.

## Data Availability

The data used to support the findings of this study are available from the corresponding authors upon reasonable request.

## Abbreviations

LBDDS: Lipid-based drug delivery system
LBFs: Lipid-based formulations
SEDDS: Self-emulsifying drug delivery system
SNEDDS: Self-nanoemulsifying drug delivery system
S-SEDDS: Solid-self-emulsifying drug delivery system
SMEDDS: Self-microemulsifying drug delivery system
SLN: Solid-lipid nanoparticle
BCS: Biopharmaceutical classification system
JNC 8: Eight Joint National Committee on prevention, detection, evaluation, and treatment
CV: Cardiovascular
CHF: Congestive heart failure
BP: Blood pressure
ABP: Arterial blood pressure
SVR: Systemic vascular resistance
RAAS: Renin-angiotensin-aldosterone system
ESH/ESC: European Society of Hypertension/European Society of Cardiology
NICE: National Institute for Health and Care Excellence
NSAID: Nonsteroidal anti-inflammatory drugs
AT 1: Angiotensin 1
AT II: Angiotensin II
ACE: Angiotensin converting enzyme
CCB: Calcium channel blocker
RDS: Rate determining step
GIT: Gastrointestinal tract
P-gp: P-glycoprotein
CYP3A4: Cytochrome 3A4
API: Active pharmaceutical ingredient
SI: Small intestine
TGs: Triglycerides
FA: Fatty acid
BS: Bile salt
PUFA: Polyunsaturated fatty acid
HPMC: Hydroxy-propyl methyl cellulose
HLB: Hydrophilic-lipophilic balance
SCF: Supercritical fluid
DLS: Dynamic light scattering
SEM: Scanning electron microscopy
TEM: Transmission electron microscopy
NLC: Nanostructured lipid carriers.
